# Polar Phenol Detection in Plasma and Serum: Insights on Sample Pre-Treatment for LC/MS Analysis and Application on the Serum of Corinthian Currant-Fed Rats

**DOI:** 10.3390/biom12121838

**Published:** 2022-12-08

**Authors:** Paraskevi B. Vasilakopoulou, Aimilia-Tatiana Gousgouni, Amalia E. Yanni, Nikolaos Kostomitsopoulos, Vaios T. Karathanos, Antonia Chiou

**Affiliations:** 1 Laboratory of Chemistry-Biochemistry-Physical Chemistry of Foods, Department of Nutrition and Dietetics, Harokopio University, 70 El. Venizelou Ave., 176 76 Kallithea, Greece; 2 Laboratory Animal Facility, Biomedical Research Foundation of the Academy of Athens, 115 27 Athens, Greece; 3 Agricultural Cooperatives’ Union of Aeghion, Corinthou 201, 251 00 Aeghion, Greece

**Keywords:** *Vitis vinifera*, Corinthian Currant, polar phenolics, LC-MS, plasma, serum, rat

## Abstract

Analysis of plasma and serum provides valuable information on the amounts of polar phenols’ circulating after ingestion. In the present study, protein precipitation (PPT), liquid–liquid extraction (LLE), solid phase extraction (SPE), enzymatic hydrolysis and their combinations were meticulously evaluated for the extraction of a variety of polar phenolic moieties from plasma and serum. The recovery values of the above methods were compared; satisfactory recoveries (>60%) were attained for most analytes. Polar phenol aglycones undergo degradation with enzymatic hydrolysis; however, their extended phase II metabolism makes enzymatic hydrolysis a mandated process for their analysis in such biofluids. Hence, enzymatic hydrolysis followed by LLE was used for the identification of polar phenols in rats’ serum, after the long-term oral consumption of Corinthian Currant. Corinthian Currant is a Greek dried vine product rich in bioactive polar phenolics. Flavonoids and phenolic acids, detected as aglycones, ranged from 0.57 ± 0.08 to 181.66 ± 48.95 and 3.45 ± 1.20 to 897.81 ± 173.96 ng/mL, respectively. The majority of polar phenolics were present as phase II metabolites, representing their fasting state in the blood stream. This is the first study evaluating the presence of polar phenolics in the serum of rats following a long-term diet supplemented with Corinthian Currant as a whole food.

## 1. Introduction

Corinthian Currants are a Mediterranean dried fruit of the vine, made from a particular variety of black grape, named *Vitis vinifera* L., var. Apyrena, which is almost exclusively cultivated in Southern Greece. One of the three commercial sub-varieties of Corinthian Currants, namely Vostizza, falls under the high-quality category and has a protected designation of origin (PDO) name. Corinthian Currants have been found to contain a plethora of polar phenolic components, including benzoic and phenyl acetic acid derivatives, hydroxy-cinnamic acids, anthocyanins and flavonoids [1,2,3,4]. While an increasing number of studies have shown a correlation between the consumption of polar phenolics and a reduction in chronic disease risk factors, discrepancies in the explanation of their beneficial effects have been found in terms of their bioavailability [5,6]. 

Indeed, polar phenolics have low bioavailability which can also vary widely among their different classes, as well as individual compounds within a given class due to several factors; the interaction with the food matrix; the metabolic processes mediated by the liver (phase I and II metabolism) and the gastrointestinal tract; and the gut microbiota [7]. Therefore, the accurate determination of their circulating blood levels, metabolites and other existing forms is imperative to aid in the understanding and confirmation of their potency and mechanisms of action. To date, most studies on animal models or human clinical trials investigate the bioavailability of polar phenolics administered by plant or fruit extracts, given alone or mixed with food [8]. For this purpose, biofluids such as plasma, serum and urine are the most commonly collected samples [5]. While they share common traits and similar components, their matrix somehow differs; as a result, these biofluids usually serve different purposes [9]. Serum is considered the gold standard; however, blot clotting could affect the levels of some constituents and the resulting serum samples are more prone to ex vivo protein degradation. On the other hand, plasma may offer a more representative image—nearly identical with circulating levels and seems to be the most common type of sample obtained for the pharmacokinetic analysis of polar phenolics; however, plasma contains more proteins than serum and various anticoagulants may cause interference during analysis, especially with mass spectrometry [10,11]. Given the above, when considering their analysis for the detection of food microconstituents in vivo, neither biofluid is clearly superior to the other; as some metabolites are found in one of the two biofluids, the one or the other biofluid may be preferred depending on the scope of research [10]. As of yet, a comparative study on the performance of plasma and serum as matrix for polar phenol determination has not been performed.

When it comes to plasma and serum samples, which both have a complex matrix and contain trace amounts of polar phenolics and their metabolites, pretreatment steps are imperative for sample purification and analyte enrichment [12]. There are numerous bioanalytical techniques available that can be applied depending on the analytical goal, available resources and the scope of the study [13]. Some of the most commonly used sample preparation methods for the simultaneous purification of plasma and serum samples and the extraction of a broad spectrum of polar phenolic moieties include protein precipitation (PPT) [14,15,16], liquid–liquid extraction (LLE) [17,18,19,20], solid phase extraction (SPE) [21,22,23], enzymatic hydrolysis [24,25,26] and combinations of the aforementioned [27,28]. Interestingly, there have been some studies aptly questioning the suitability of enzymatic hydrolysis for the analysis of polar phenolics in biospecimen which we also took into account for the present work [24,29]. Enzymatic hydrolysis is a very common analytical sample pre-treatment method, used to quantify glucuronidated and/or sulfated forms of polar phenolics, i.e., conjugated phase II metabolites, in biofluids [9]. Due to the lack of commercially available pure standards and the difficulty in synthesizing the aforementioned metabolites, enzymatic hydrolysis is the easiest solution for many clinical studies exploring the bioavailability of polar phenolics in vivo. The most frequently used enzyme for polar phenolics is from *Helix pomatia*, which contains β-glucuronidase and a smaller amount of sulfatase [30]. The enzymes used in this case break the O-glucuronide or O-sulfate bonds, releasing the polar phenolic precursor, also known as free form polar phenol or aglycone, which is then quantified by employing analytical methods such as gas chromatography coupled to a mass spectrometer (GC-MS) [3,31] and liquid chromatography coupled to a mass spectrometer or a diode array detector [9,32,33].

Within this context, we aimed to apply some of the most commonly used sample preparation methods for plasma and serum samples. On this basis, ten sample preparation procedures were compared in each case; PPT with organic solvents with or without the addition of acid; hybrid methods combining the aforementioned PPT conditions and SPE; LLE with ethyl acetate with or without the addition of acid prior extraction. In addition, the implementation of enzymatic hydrolysis with *Helix pomatia*, prior to liquid–liquid extraction with EtOAc of serum and plasma samples, was evaluated. The % extraction recoveries (% R), which served as the main evaluation index, were obtained by comparing spiked versus matrix-matched plasma and serum samples in each case. Ultra-high performance liquid chromatography coupled to mass spectrometry (UHPLC-MS) was used for the detection and quantification of the studied polar phenolic moieties [4]. 

With respect to Corinthian Currant polar phenols, studies evaluating their presence in biological fluids after the consumption of this food as a whole are scarce [3,31], while no data exist on their presence in serum after a long term intervention study. The optimized method proposed herein was applied for the identification of polar phenols in rats’ serum, after they have followed a long-term Corinthian Currant supplemented daily diet. In this framework, the existence of polar phenols in rat serum was evaluated and compared among animal groups consuming, or not, Corinthian Currants for 28 days. 

## 2. Materials and Methods

### 2.1. Reagents, Chemicals and Materials

(−)-Epicatechin, (−)-epigallocatechin, (+)-catechin, apigenin, chrysin, luteolin, kaempferol, (±)-hesperetin, (±)-naringenin, daidzein, formononetin, genistein, trans-resveratrol, 3-hydroxytyrosol, chlorogenic acid, ferulic acid, neochlorogenic acid, p-coumaric acid, sinapic acid, syringic acid and trans-cinnamic acid were purchased from Supelco (Bellefonte, PA, USA); epicatechin gallate, (+)-E-viniferin, caffeic acid, gallic acid, o-coumaric acid, and vanillic acid were purchased from Sigma-Aldrich, Inc. (St. Louis, MO, USA); (−)-epigallocatechin gallate, 3-O-methylquercetin (isorhamnetin), quercetin, quercetin-3-O-glucoside (isoquercetin) and quercetin-3-O-rutinoside (rutin) were purchased from Extrasynthese (Genay, France); piceid was purchased from Fluorochem (Hadfield, UK) and an isotopically labeled internal standard (IS), quercetin d-3, was purchased from Cayman Chemical (Ann Arbor, MI, USA). Optima™ LC/MS grade formic acid 99.0%+, used as an ultra-pure additive in the mobile phase, along with LC/MS grade solvents (acetonitrile (ACN), methanol (MeOH) and water (H_2_O)) and HPLC grade ethyl acetate (EtOAc), were purchased from Thermo Fisher Scientific (Pittsburgh, PA, USA). Phosphoric acid (H_3_PO_4_), sodium acetate (CH_3_COONa) and acetate acid (CH_3_COOH) were obtained from Thermo Fisher Scientific (Waltham, MA, USA). Oasis^®^ HLB (3 cc/60 mg) was obtained from Waters (Milford, MA, USA). Sulfatase and β-glucuronidase from *Helix pomatia*—Type H-2, aqueous solution, sulfatase ≥ 2000 U/mL and glucuronidase ≥ 100,000 U/mL, were purchased from Sigma-Aldrich (St. Louis, MO, USA).

Vostizza Corinthian Currants were provided by the Agricultural Cooperatives’ Union of Aeghion, Greece.

### 2.2. Preparation of Stock and Working Solutions

Working standard solution mixtures were prepared by appropriately diluting stock standard solutions of the analytes (1 mg/mL) in MeOH. Internal standard (IS) stock solution (0.5 mg/mL), namely quercetin-d3, was also prepared in MeOH, while working solutions of IS were prepared at a concentration of 0.5 μg/mL. All other solvent and matrix-matched solutions were prepared by diluting working standard solutions in MeOH-H_2_O (1:1, *v*/*v*) and blank EDTA-plasma/serum samples, respectively, along with the addition of IS (50 ng/mL). Quality control samples were prepared accordingly. All samples and standard solutions were kept at −40 °C.

### 2.3. Instrumentation Conditions

The chromatographic analysis was carried out on a Dionex UltiMate™ 3000 Rapid Separation UHPLC+ (Thermo Fisher Scientific, Bremen, Germany) coupled to an Exactive Plus™ Orbitrap mass spectrometer (Thermo Fisher Scientific, Bremen, Germany), equipped with a heated electrospray ionization probe (HESI-II; Thermo Fisher Scientific, USA) operated in negative mode. The chromatographic conditions and mass spectrometry parameters for the analysis of polar phenolics were set as previously described by Vasilakopoulou et al. [4]. The total run time was 18 min. Quantitative and qualitative data processing was performed using TraceFinder ™ software (Version 4.1, Thermo Fisher Scientific, San Jose, CA, USA). Chromatographic and mass spectral data for each analyte and the internal standard (IS) are given in Table S1.

### 2.4. Sample Preparation

Ten sample preparation methods were compared in the present work in order to evaluate the extraction performance of polar phenolics from both plasma and serum. These procedures included protein precipitation (PPT), PPT followed by solid phase extraction (SPE), liquid–liquid extraction (LLE) and enzymatic hydrolysis followed by LLE. 

#### 2.4.1. Deproteinization with ACN or MeOH/ACN 1:9

ACN or a mixture of MeOH/ACN 1:9 (400 μL) was added to plasma/serum (100 μL) and centrifuged for 15 min at 7000× *g*. The supernatant was collected, evaporated and the residue was reconstituted in 100 μL MeOH-H_2_O (1:1, *v*/*v*) for LC-MS analysis. The same steps were followed with the same solvents acidified (0.1% HCOOH).

#### 2.4.2. Deproteinization with ACN or MeOH/ACN 1:9, followed by SPE

The plasma/serum samples were initially treated as described in Section 2.4.1. After evaporation, the residues were reconstituted in 400 μL of 0.1% (*v*/*v*) HCOOH and H_2_O, and were loaded onto OASIS^®^ HLB SPE cartridges as described by Vasilakopoulou et al. [4]. Briefly, 1.5 mL of MeOH followed by the same volume of 0.1% formic acid were used to precondition the sorbent of the SPE cartridges. Afterwards, the aforementioned residues were loaded and then the SPE cartridges were washed with 1.5 mL of 0.1% formic acid; the retained analytes were eluted with 4 mL of MeOH. The eluents were then collected, evaporated and reconstituted as described in Section 2.4.1.

#### 2.4.3. Liquid–Liquid Extraction with EtOAc

EtOAc (700 μL) were added to plasma/serum samples and were vortexed. Subsequently, the samples were centrifuged for 5 min at 5000× *g* and the supernatants were collected. The extraction was performed a total of three times and the supernatants were combined. The obtained extracts were evaporated and reconstituted as described in Section 2.4.1. The same steps were followed after the acidification of plasma/serum samples with HCOOH (1% *v*/*v*).

#### 2.4.4. Enzymatic Hydrolysis Followed by LLE with EtOAc

CH_3_COONa (100 μL, 0.1 M, pH 5) were added to plasma/serum samples (100 μL). β-glucuronidase, 2000 U and sulfatase, 40 U (20 μL) were added to the samples which were vortexed and then incubated for 45 min at 37 °C in a heating bath. H_3_PO_4_ (100 μL, 4% *v*/*v*) was added to terminate the enzymatic hydrolysis. Subsequently, the samples were treated with LLE as described in Section 2.4.3.

### 2.5. Method Validation

Method validation was conducted in accordance with the European Medicines Agency (EMA) 2011 guidelines on bioanalytical method validation [34]. 

#### 2.5.1. Specificity and Selectivity

Blank serum/plasma samples (control), blank serum/plasma samples containing only IS (zero), and blank serum/plasma samples spiked with standard solutions of the studied analytes and IS (spiked) were analyzed for the evaluation of method specificity and selectivity. 

#### 2.5.2. Linearity, Carry-Over and Matrix Effect

Solvent and matrix-matched calibration curves for all analytes were obtained and fitted by least-squares linear regression. Limits of detection (LODs) and limits of quantification (LOQs) were calculated based on the standard deviation of the response and the slope of the calibration curve obtained using a range of low values close to zero. Blank samples were injected right after the upper LOQ sample for the carry-over assessment. Carry-over was regarded as insignificant if the measured peak areas were below 20% of the lowest limit of quantification (LLOQ) of the analytes and 5% of the IS. The matrix effect (ME %) was evaluated by comparing the slopes of standard curves dissolved in extraction solvent and matrix-matched calibration curves, and was calculated as follows: 100 × [(matrix slope)/(solvent slope)−1] (Table S2).

#### 2.5.3. Precision and Accuracy

Intra- and inter-day precision (% RSD) and accuracy (% RE) were assessed by analyzing quality control (QC) samples at various levels (0.5–160 ng/mL depending on the compound) prepared in five replicates. Precision and accuracy values within ± 15% (± 20% for LLOQ) were considered acceptable (Table S3).

#### 2.5.4. Stability

QC samples were analyzed after preparation and after the applied storage conditions that were to be evaluated. The QC samples were also analyzed against a calibration curve and the obtained concentrations were compared to the nominal concentrations. The mean concentration at each assayed level was set within ± 15% of the nominal concentration (Table S4).

#### 2.5.5. Extraction Recovery

The extraction recovery was evaluated by calculating the peak responses of spiked matrix samples and comparing them with those obtained from the matrix-matched samples at the corresponding amounts.

### 2.6. Polar Phenol Content of Corinthian Currant and Rat Chow

Polar phenols were extracted from Corinthian Currants and rat chow with MeOH as described by Chiou et al. [1]. Briefly, the solvent was evaporated under vacuo and the residues were reconstituted in MeOH (2 mL). Aliquots (200 μL) of the crude polar phenol extracts were further treated with SPE using Oasis^®^ HLB cartridges, as described by Vasilakopoulou et al. [4]. 

### 2.7. Animals and Diet 

Twelve male RccHan^®^: WIST rats (350–400 g bw) were randomized into two groups (six animals per group) according to their dietary regimen, i.e., healthy animals that received the control diet, i.e., rat chow (C) or control diet supplemented with 10% *w*/*w* Corinthian Currant (CC) for 4 weeks. At the end of the intervention, animals were sacrificed and blood samples were collected by cardiac puncture; serum samples were isolated and stored at −80 °C until analysis. Animal experimentation was reviewed and approved by the Veterinary Directorate of the Athens Prefecture (Ref. Number 453264/07-08-2019), and conducted in compliance with the European Directive 2010/63. Animal housing took place in the Centre of Experimental Surgery of the Biomedical Research Foundation of the Academy of Athens (BRFAA). The animals were housed in accordance with the European legal framework and the international guidelines existing for the protection of animals used for scientific purposes.

### 2.8. Statistical Analysis

All statistical analyses were performed using IBM SPSS Statistics for Windows, version 26 (IBM Corp., Armonk, NY, USA). For multiple comparisons among samples subjected to different sample treatment procedures, one-way analysis of variance (ANOVA) was used. Tukey’s multiple range tests were performed post hoc to evaluate differences among groups. Comparisons for the detected levels of polar phenolics were conducted with dependent samples T-test for the comparisons within the control (C vs. Cenz) and the dietary intervention group (CC vs. CCenz), whereas independent samples T-tests were applied for the comparisons among the control and dietary intervention group (C vs. CC, and Cenz vs. CCenz). Data are presented as mean ± standard deviation (SD). The level of statistical significance was set at *p* < 0.05.

## 3. Results and Discussion

### 3.1. Extraction Performance Comparison of Sample Preparation Methods

In the present study, in order to assess the extraction performance of polar phenolics from both plasma and serum, ten sample preparation procedures were compared in each case; PPT with organic solvents with or without the addition of acid; hybrid methods combining the aforementioned PPT conditions and SPE; LLE with ethyl acetate with or without the addition of acid prior extraction. The % extraction recoveries (% R), which served as the main evaluation index, were obtained by comparing spiked versus matrix-matched plasma and serum samples, at a concentration level of 100 ng/mL for all studied compounds (Table 1 and Table 2).

The most generic method to prepare plasma and serum samples in large scale studies is protein precipitation (PPT), by adding 3–5 times the sample’s volume of organic solvents such as acetonitrile, methanol, ethanol or mixtures of the aforementioned, and/or acidified solutions depending on the stability of analytes in low pH [35]. Even though PPT is considered a rapid and simple method, it is a non-selective technique offering poor sample cleanup, as major endogenous substances such as phospholipids tend to coelute with the analytes, leading to interferences during chromatographic separation and ionization in the mass spectrometer [36,37]. In the present study, both plasma and serum samples that underwent the PPT methods had the lowest % recoveries; the majority of the studied analytes attained % recoveries ranging around 40%. Similarly, the use of acidified solvents did not seem to lead to any improvements in extraction recovery (Table 1 and Table 2).

In contrast, the hybrid methods combining PPT and SPE seemed to improve the extraction recoveries for most of the analytes from both studied biofluids; the extraction recoveries of most polar phenolics ranged from 40–80%, with a fewer being > 80% (Table 1 and Table 2). This is probably attributed to better analyte ionization under the ESI conditions employed. However, this finding was not uniform among the different PPT extraction solvents employed in the present study. In particular, the combination of acidified extraction solvents for PPT and SPE seemed to be more favorable in the case of plasma samples when compared to the respective serum samples; the extraction recoveries of the majority of analytes in plasma samples ranged from 60% or higher, whereas in the serum, the extraction recoveries ranged for the majority of analytes from 40–60%, with fewer being higher than 60%. In any case, it is apparent that SPE improved the extraction recovery of polar phenolics from plasma and serum samples. 

Liquid–liquid extraction (LLE) with ethyl acetate (EtOAc) in non-acidified plasma and serum samples yielded rather high extraction recoveries for the majority of polar phenolics; almost all had extraction recoveries > 60%, of which most varied from 70–105%. The extraction recoveries from LLE were similar to the PPT + SPE treated samples for most analytes, especially for flavonols, flavones, isoflavones and most phenolic acids in the case of serum samples. Acidification of plasma and serum samples prior to LLE did not seem to improve analyte extraction; on the contrary, this method led to extraction recoveries < 60%. Overall, our results indicate that LLE with EtOAc and no prior sample acidification led to somewhat satisfactory recoveries, consumed less time for sample preparation and is a more affordable alternative than PPT + SPE. Hence, LLE with EtOAc and no acidification was chosen for the ensuing analyses for both plasma and serum samples.

### 3.2. Extraction Performance of Enzymatic Hydrolysis, Followed by LLE

Prior to liquid–liquid extraction with EtOAc, serum and plasma samples underwent enzymatic hydrolysis treatment. The hydrolysis protocol performed was based on previously published studies [29,31]. In this context, blank serum and plasma samples were spiked with a mix standard solution of polar phenolics (100 ng/mL). In order to assess the recovery of polar phenolics in their free form, four control samples were prepared using the same spiked serum and plasma samples; the first control underwent the same procedure but without the addition of enzyme to evaluate whether the temperature (37 °C) and pH (5.0) conditions used for the hydrolysis affected the recoveries (Table 3, (−)), while the second control underwent the same procedure with the addition of enzymes (Table 3, (+)). The third control sample was prepared without following the hydrolysis procedure while the fourth control sample was a matrix-matched sample. The analytes’ recoveries from the aforesaid spiked serum and plasma samples were compared to their respective matrix-matched samples.

According to our results (Table 3), the temperature (37 °C) and pH (5.0) affected the recovery of only a few of the polar phenolics, given that these conditions are considered mild. The enzyme addition resulted in a significantly lower recovery of almost all compounds (*p* < 0.05), with the glycosides in particular suffering a notable reduction of almost 100% in both plasma and serum samples. Some flavonoids, namely epigallocatechin, epigallocatechin gallate, naringenin, kaempferol, isorhamnetin and quercetin also experienced a significant reduction of up to 85% under these conditions. These findings are in line with [29], who also reported that the recovery of naringenin and quercetin was negatively affected by the presence of enzymes; the decrease in their recoveries was also found to be significantly correlated with the increase in enzymatic concentration. Even though the enzymatic concentration used in the present study (2000 U β-glucuronidase/40 U sulfatase) was lower than the lowest concentration of enzymes used in the study of [29] (3493 U β-glucuronidase/129 U sulfatase), a comparable degradation of free polar phenolics was noted. However, given that most polar phenolics after ingestion are present in the blood stream mainly as glucuronidated and sulfated conjugates, and not in their aglycone form [32], the application of enzymatic hydrolysis in this case should have minimal negative effect.

### 3.3. Polar Phenolics Detected in Serum of Corinthian Currant-Fed Rats

**Figure 1:**
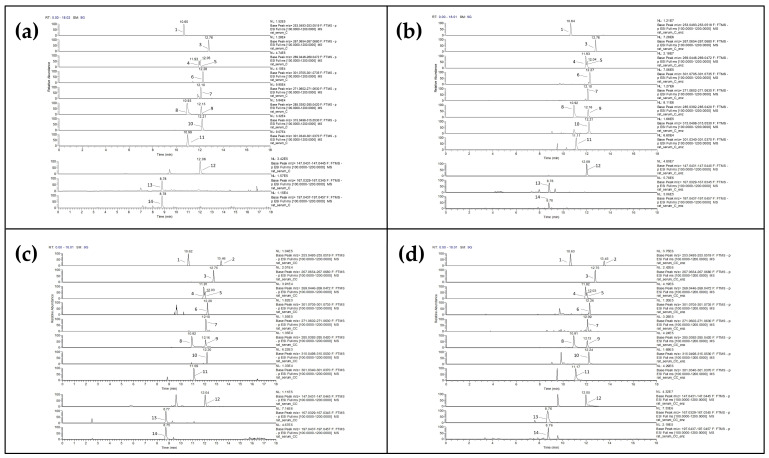
Extracted ion chromatograms (XICs) of polar phenolic compounds detected in rat serum from (**a**) the control group, (**b**) the control group that underwent enzymatic hydrolysis, (**c**) the Corinthian Currant supplemented group and (**d**) the Corinthian Currant supplemented group that underwent enzymatic hydrolysis. Peaks: (1) daidzein, (2) chrysin, (3) formononetin, (4) apigenin, (5) genistein, (6) hesperetin, (7) naringenin, (8) luteolin, (9) kaempferol, (10) isorhamnetin, (11) quercetin, (12) trans-cinnamic acid, (13) vanillic acid and (14) syringic acid.

By adopting a simple serum treatment method that included the enzymatic hydrolysis of samples followed by liquid–liquid extraction (LLE) with EtOAc, we were able to detect and quantify polar phenolics that were present in the rats’ serum as a result of their diet. In our study, we evaluated the content of free and conjugated forms of various flavonoids and phenolic acids in both intervention groups (C and CC); β-Glucuronidase/sulfatase from *Helix pomatia* was used to hydrolyze the conjugates to form free polar phenolics, i.e., their aglycones, which were measured with the present LC-MS method. A typical chromatogram (XIC) of rat serum is given in Figure 1. Rats’ blood was collected after overnight fasting; the detected and quantified polar phenolics represent their fasting state in serum. Unsurprisingly, polar phenolics were detected in both groups (C and CC) of rats’ serum samples. Given that the rat chow is primarily comprised of plant-based ingredients including soy, barley and forage, it is relatively expected that the rats’ serum of the control group (C) would contain polar phenolics [4]. To the best of our knowledge, this is the first long-term study evaluating the existence of polar phenolics in the serum of rats following their typical diet (C, Cenz) or a long-term diet supplemented with 10% (*w*/*w*) Corinthian Currant as a whole food (CC, CCenz).

#### 3.3.1. Comparisons of C vs. CC and Cenz vs. CCenz

Analysis with independent T-tests revealed statistically significant differences among the detected levels of free polar phenolics among the two intervention groups; the Corinthian Currant supplemented group showed a tendency towards higher levels of the detected free polar phenolics when compared to the non-supplemented group (Table 4). When comparing the samples of the control (C) and the dietary intervention group (CC), the detected flavonoids were below their limits of quantification, with the exception of apigenin, daidzein, luteolin and formononetin. The flavonoids apigenin, daidzein and formononetin were found at statistically significantly higher levels in the CC group; their corresponding amounts were 1.53 ± 0.36 (*p* = 0.006), 4.35 ± 1.62 (*p* = 0.039) and 1.85 ± 0.54 (*p* = 0.005) ng/mL, respectively. Luteolin was detected and quantified in both groups (C & CC); however, with no statistically significant differences (*p* = 0.297). Phenolic acids were also detected at quantifiable amounts in both serum samples of the control (C) and dietary intervention group (CC); vanillic acid was detected at statistically significantly higher amounts in the CC group compared to the C group (*p* = 0.017). 

When comparing the enzymatically treated serum samples of the control and dietary intervention group (Cenz and CCenz, respectively), the flavonoids apigenin, chrysin, luteolin, isorhamnetin, quercetin, hesperetin and genistein were found at statistically significantly higher amounts in the CCenz group; their detected levels were 181.66 ± 48.95 (*p* = 0.046), 2.54 ± 1.06 (*p* = 0.005), 113.74 ± 66.08 (*p* < 0.000), 5.93 ± 2.22 (*p* = 0.016), 8.54 ± 4.83 (*p* = 0.004), 9.80 ± 3.82 (*p* = 0.036) and 40.40 ± 16.28 (*p* = 0.049) ng/mL, respectively. In the study of Yanni et al. [31], the flavonoids catechin and chrysin, as well as several phenolic acids, were found in the fasting plasma of the control and the Corinthian Currant-fed New Zealand white rabbits; solely p-hydroxybenzoic acid was found at a higher content in the intervention group after eight weeks. The content of these metabolites is one to two orders of magnitude lower than those found in our study, with the exception of chrysin; the variations observed may be attributed to differences in the animal model, the biological fluid evaluated, as well as the extraction and enzymatic hydrolysis protocols applied. 

#### 3.3.2. Comparisons of C vs. Cenz and CC vs. CCenz

Analysis with dependent-samples T-tests revealed statistically significant differences among the detected levels of free polar phenolics in the rats’ serum samples that underwent enzymatic hydrolysis prior to LLE compared to the ones that were not treated with enzymatic hydrolysis, in both intervention groups. For almost all detected polar phenolics, the enzymatically incubated serum samples prior to LLE treatment yielded statistically significantly higher levels of their free forms (Table 4). This is consistent with earlier research that showed that different polar phenolics undergo substantial conversion into sulfated and glucuronidated conjugates after ingestion, and that their aglycone forms could not be detected in plasma samples that are not treated with enzymatic hydrolysis (Ding et al., 2013). In this context, it is of particular interest that in both intervention groups and especially after the long-term supplementation of Corinthian Currant, flavonoids and phenolic acids seem to be present as glucuronate or sulfate conjugates in the rats’ serum after overnight fasting.

As the suitable conjugate standards of polar phenolics are rarely commercially available for analysis, the majority of studies on their bioavailability repeatedly treat biofluid samples with glucuronidase/sulfatase enzymes for the subsequent quantification of the released aglycones [38]. To date, a few studies have questioned the efficacy of enzymatic hydrolysis for the reliable quantification of glucuronidated and sulfated metabolites of polar phenolics in biofluids [24,29,39]. According to Quifer-Rada et al. [29], enzymatic hydrolysis negatively affected the recovery of the precursor and free-form polar phenolics present in human urine samples. Stohs et al. [24] suggest that the use of enzymatic hydrolysis of plasma samples greatly exaggerates the amount of free polar phenolics detected, resulting in an actual determination of the free plus conjugated form of polar phenolics. On another note, Luis et al. [39], by comparing the efficiencies of β-glucuronidase and sulfatase from *Helix pomatia* to hydrolyze curcumin conjugates in mouse plasma after oral administration of turmeric, suggested that the first leads to incomplete hydrolyzation of complex sulfate conjugates, which could result in an underestimation of the total plasma concentration of curcumin. 

Considering our results on the effect of enzymatic hydrolysis on free polar phenolics and all of the aforesaid, while this methodology seems to offer helpful insights, it is crucial to keep in mind that this type of information is indirect and offers a quantitative estimate of the polar phenol aglycones in vivo levels. That being said, without knowing the exact nature and different proportions of polar phenolic conjugates and sites of conjugation that may be present in the blood as a result of the ingested food source and the characteristics of the host, it is hard to know the exact mechanisms that take place in serum during hydrolysis and how they may affect their absorption and consequently their detection and quantitation [30,40]. 

The results of our study may offer some insight on the circulating levels of polar phenolics, which in return indicate that the observed rise in some polar phenolics could be a result of consuming Corinthian Currant on a daily basis over a prolonged period of time. Flavanones and isoflavones have been shown to be among the flavonoids with the best bioavailability profiles; for instance, plasma concentrations in adults who consumed relatively low levels of soya derivatives supplying approximately 50 mg of isoflavones reached 1–4 μM even after 6–8 h [40]. In our case, it appears that the rat chow contains higher amounts of isoflavones (daidzein, formononetin and genistein), somewhat similar levels of flavanones (hesperetin and naringenin) and higher amounts of some flavones (apigenin and luteolin) than Corinthian Currants (Table S5). This could be seen as a limitation of our study, given that a polar phenol-free diet for the rats is largely inevitable as their feed is plant-based. Hence, the bioavailability of the polar phenols of Corinthian Currant in the diet under investigation may be impacted to some extent by the rat chow. 

The understanding of bioavailability is incredibly challenging since natural foods are comprised of intricate mixes of several free and conjugated polar phenolics, each of which has a unique bioavailability, metabolism and excretion profile. Moreover, some phenolic acids are also present in vivo as byproducts of physiological metabolic pathways. Furthermore, low molecular weight phenolic compounds such as various benzoic, phenylacetic, and propionic acids are the main microbiota catabolites produced after the intake of flavonoids and anthocyanins [41].

## 4. Conclusions

LLE extraction with ethyl acetate along with PPT, via acidified solvents that is followed by SPE, were proven to be the most efficient sample preparation protocols for the analysis of plasma and serum polar phenols. Nevertheless, LLE provides advantages with respect to time and labor. Plasma or serum may be used, yielding rather similar recoveries; the selection may be actually based on the scope of each study. Enzymatic hydrolysis with β-glucuronidase/sulfatase from *Helix pomatia*—Type H-2, seemed to have a negative effect on the polar phenol aglycones, adding to prior research questioning this widely used analytical strategy. However, the extended phase II metabolism of polar phenols makes enzymatic hydrolysis an imposed procedure for their analysis in plasma or serum.

Following a long-term Corinthian Currant supplemented diet, polar phenolics were detected and quantified in both groups (C and CC) of rats’ serum, representing their fasting state in the blood stream. The majority of detected flavonoids and phenolic acids were present as phase II metabolites, given that after the implementation of enzymatic hydrolysis their aglycones were found at quantifiable amounts. Our findings may shed light on the amounts of polar phenolics in circulation, which in return indicate that the observed increase of some polar phenolics may be linked to the regular consumption of Corinthian Currant as a whole food over an extended period of time.

## Figures and Tables

**Table 1:** Recovery (%R) data of the studied polar phenols after testing ten different sample preparation methods for plasma (n = 3).

Analyte	PPT_1	PPT_2	PPT_3	PPT_4	PPT + SPE_1	PPT + SPE_2	PPT + SPE_3	PPT + SPE_4	LLE_1	LLE_2
Isoflavones										
Daidzein	55 ± 3 ^ab^	48 ± 0 ^ab^	38 ± 2 ^a^	41 ± 2 ^a^	47 ± 3 ^ab^	116 ± 6 ^d^	115 ± 9 ^d^	61 ± 18 ^b^	90 ± 8 ^c^	56 ± 4 ^ab^
Formononetin	54 ± 2 ^bc^	45 ± 0 ^ab^	32 ± 1 ^a^	33 ± 1 ^a^	42 ± 4 ^ab^	118 ± 9 ^e^	110 ± 6 ^e^	69 ± 20 ^c^	84 ± 7 ^d^	50 ± 2 ^b^
Genistein	52 ± 2 ^b^	47 ± 1 ^ab^	34 ± 1 ^a^	39 ± 2 ^ab^	36 ± 3 ^ab^	82 ± 3 ^c^	86 ± 5 ^c^	74 ± 22 ^c^	84 ± 11 ^c^	52 ± 3 ^b^
Flavanones										
Hesperetin	45 ± 2 ^b^	44 ± 0 ^ab^	27 ± 1 ^a^	35 ± 2 ^ab^	38 ± 3 ^ab^	109 ± 7 ^d^	80 ± 6 ^c^	75 ± 22 ^c^	87 ± 6 ^cd^	51 ± 3 ^b^
Naringenin	47 ± 2 ^bc^	47 ± 1 ^bc^	26 ± 1 ^a^	37 ± 1 ^abc^	33 ± 3 ^ab^	106 ± 7 ^ef^	78 ± 6 ^de^	107 ± 8 ^f^	83 ± 9 ^d^	53 ± 3 ^c^
Flavones										
Apigenin	33 ± 2 ^a^	40 ± 0 ^ab^	26 ± 1 ^a^	28 ± 1 ^a^	98 ± 7 ^d^	86 ± 12 ^cd^	74 ± 8 ^c^	87 ± 9 ^c^	80 ± 12 ^c^	50 ± 3 ^b^
Chrysin	47 ± 1 ^b^	45 ± 1 ^b^	20 ± 1 ^a^	21 ± 0 ^a^	98 ± 10 ^d^	84 ± 11 ^c^	77 ± 7 ^c^	77 ± 6 ^c^	78 ± 8 ^c^	51 ± 3 ^b^
Luteolin	28 ± 2 ^a^	34 ± 1 ^ab^	26 ± 1 ^a^	27 ± 0 ^a^	66 ± 4 ^c^	76 ± 11 ^cd^	75 ± 5 ^cd^	95 ± 7 ^d^	76 ± 13 ^cd^	46 ± 8 ^b^
Flavonols										
Isorhamnetin	n.d.	27 ± 15 ^b^	25 ± 0 ^b^	25 ± 1 ^b^	42 ± 3 ^c^	56 ± 11 ^d^	77 ± 5 ^e^	84 ± 6 ^e^	80 ± 8 ^e^	59 ± 3 ^d^
Kaempferol	5 ± 1 ^a^	n.d.	22 ± 0 ^b^	24 ± 1 ^b^	92 ± 7 ^f^	58 ± 13 ^cd^	67 ± 6 ^de^	80 ± 7 ^e^	77 ± 10 ^e^	57 ± 3 ^c^
Quercetin	n.d.	7 ± 7 ^a^	22 ± 0 ^b^	21 ± 0 ^b^	6 ± 6 ^a^	55 ± 14 ^c^	69 ± 5 ^de^	75 ± 4 ^de^	80 ± 10 ^e^	63 ± 10 ^cd^
Flavan-3-ols										
Catechin	n.d.	n.d.	n.d.	n.d.	32 ± 2 ^b^	55 ± 10 ^c^	62 ± 6 ^cd^	59 ± 2 ^cd^	66 ± 9 ^a^	4 ± 2 ^d^
Epicatechin	n.d.	n.d.	n.d.	n.d.	67 ± 4 ^cd^	56 ± 11 ^b^	62 ± 5 ^bc^	60 ± 1 ^bc^	73 ± 7 ^d^	1 ± 1 ^a^
Epicatechin gallate	n.d.	n.d.	n.d.	n.d.	n.d.	61 ± 15 ^c^	72 ± 5 ^c^	59 ± 3 ^c^	91 ± 16 ^d^	24 ± 1 ^b^
Epigallocatechin	n.d.	n.d.	n.d.	n.d.	n.d.	50 ± 11 ^b^	57 ± 5 ^bc^	61 ± 2 ^c^	71 ± 15 ^c^	2 ± 1 ^a^
Epigallocatechin gallate	n.d.	n.d.	n.d.	n.d.	n.d.	55 ± 16 ^c^	65 ± 5 ^c^	55 ± 3 ^c^	92 ± 9 ^d^	18 ± 4 ^b^
Procyanidin B2	n.d.	n.d.	n.d.	n.d.	n.d.	52 ± 14 ^c^	59 ± 4 ^c^	44 ± 2 ^b^	n.d.	n.d.
Flavonol glycosides										
Isoquercetin	30 ± 4 ^ab^	28 ± 2 ^ab^	31 ± 2 ^ab^	34 ± 2 ^b^	18 ± 2 ^a^	86 ± 6 ^d^	68 ± 11 ^d^	72 ± 6 ^d^	105 ± 12 ^e^	51 ± 2 ^c^
Rutin	32 ± 2 ^ab^	29 ± 3 ^ab^	29 ± 1 ^ab^	36 ± 2 ^b^	21 ± 3 ^a^	93 ± 5 ^d^	73 ± 4 ^c^	65 ± 8 ^c^	99 ± 8 ^d^	66 ± 3 ^c^
Stilbenes										
*trans*-Resveratrol	37 ± 3 ^c^	n.d.	4 ± 1 ^a^	15 ± 2 ^b^	28 ± 2 ^c^	60 ± 6 ^de^	48 ± 5 ^d^	62 ± 4 ^ef^	69 ± 9 ^f^	61 ± 2 ^ef^
E-Viniferin	37 ± 2 ^c^	23 ± 1 ^bc^	12 ± 1 ^ab^	23 ± 1 ^bc^	n.d.^a^	67 ± 20 ^d^	63 ± 13 ^d^	69 ± 10 ^d^	66 ± 10 ^d^	98 ± 6 ^e^
Piceid	51 ± 2 ^de^	43 ± 1 ^cd^	22 ± 4 ^ab^	32 ± 2 ^bc^	12 ± 2 ^a^	79 ± 5 ^g^	62 ± 5 ^fg^	95 ± 11 ^h^	99 ± 8 ^h^	59 ± 4 ^ef^
Phenylethanoids										
3-Hydroxytyrosol	14 ± 2 ^a^	6 ± 0 ^a^	36 ± 2 ^bc^	39 ± 2 ^bc^	34 ± 7 ^b^	99 ± 8 ^g^	68 ± 3 ^ef^	59 ± 16 ^de^	76 ± 6 ^f^	49 ± 2 ^cd^
Oleuropein	n.d.	n.d.	n.d.	39 ± 1 ^bc^	33 ± 11 ^b^	56 ± 11 ^de^	74 ± 4 ^f^	67 ± 2 ^ef^	95 ± 12 ^g^	50 ± 4 ^cd^
Cinnamic acid and derivatives										
Caffeic acid	n.d.	n.d.	30 ± 3 ^b^	39 ± 1 ^bc^	51 ± 9 ^c^	53 ± 9 ^c^	65 ± 10 ^d^	109 ± 2 ^e^	87 ± 9 ^d^	50 ± 2 ^c^
*trans*-Cinnamic acid	64 ± 5 ^def^	45 ± 3 ^bc^	20 ± 2 ^a^	29 ± 2 ^ab^	45 ± 16 ^bc^	105 ± 11 ^g^	75 ± 5 ^ef^	60 ± 15 ^cde^	82 ± 6 ^fg^	46 ± 2 ^bcd^
Chlorogenic acid	24 ± 1 ^ab^	3 ± 1 ^a^	33 ± 6 ^b^	55 ± 4 ^cd^	68 ± 7 ^d^	100 ± 8 ^f^	65 ± 5 ^de^	103 ± 3 ^ef^	41 ± 3 ^bc^	26 ± 2 ^b^
*o*-Coumaric acid	49 ± 1 ^ab^	42 ± 1 ^a^	36 ± 2 ^a^	39 ± 1 ^a^	42 ± 8 ^a^	80 ± 4 ^c^	108 ± 8 ^d^	64 ± 17 ^b^	93 ± 7 ^cd^	47 ± 2 ^a^
*p*-Coumaric acid	48 ± 2 ^bc^	43 ± 1 ^abc^	33 ± 3 ^a^	39 ± 2 ^ab^	51 ± 4 ^c^	77 ± 4 ^d^	69 ± 11 ^de^	84 ± 6 ^ef^	88 ± 5 ^f^	51 ± 2 ^c^
Ferulic acid	48 ± 2 ^c^	42 ± 0 ^bc^	28 ± 4 ^ab^	39 ± 1 ^abc^	23 ± 3 ^a^	80 ± 5 ^d^	64 ± 9 ^d^	117 ± 2 ^e^	88 ± 5 ^d^	51 ± 3 ^c^
Neochlorogenic acid	20 ± 1 ^ab^	3 ± 1 ^a^	27 ± 6 ^b^	52 ± 3 ^c^	17 ± 5 ^ab^	79 ± 9 ^d^	72 ± 7 ^d^	107 ± 11 ^d^	25 ± 7 ^ab^	33 ± 3 ^bc^
Sinapic acid	47 ± 1 ^bc^	42 ± 1 ^bc^	29 ± 4 ^ab^	37 ± 2 ^ab^	20 ± 2 ^a^	87 ± 6 ^e^	62 ± 8 ^de^	120 ± 11 ^f^	85 ± 5 ^e^	56 ± 3 ^cd^
Benzoic acid derivatives										
Gallic acid	n.d.	n.d.	32 ± 2 ^b^	33 ± 1 ^b^	6 ± 1 ^a^	40 ± 7 ^bc^	62 ± 12 ^d^	58 ± 17 ^c^	38 ± 7 ^bc^	78 ± 7 ^d^
Syringic acid	n.d.	n.d.	33 ± 3 ^bc^	23 ± 1 ^ab^	n.d.	22 ± 7 ^b^	58 ± 12 ^cd^	11 ± 8 ^ab^	76 ± 9 ^d^	24 ± 16 ^b^
Vanillic acid	56 ± 3 ^bc^	49 ± 1 ^abc^	39 ± 2 ^a^	37 ± 1 ^a^	44 ± 15 ^ab^	81 ± 5 ^d^	77 ± 6 ^d^	59 ± 11 ^c^	86 ± 11 ^d^	59 ± 3 ^c^

Data are presented as mean ± SD. Treatments as follows: Protein precipitation with ACN (PPT_1) or MeOH/ACN 1:9 (PPT_2) or ac. ACN (PPT_3) or ac. MeOH/ACN 1:9 (PPT_4), protein precipitation with ACN followed by SPE (PPT + SPE_1) or MeOH/ACN 1:9 followed by SPE (PPT + SPE_2) or ac. ACN followed by SPE (PPT + SPE _3) or ac. MeOH/ACN 1:9 followed by SPE (PPT + SPE _4), liquid–liquid extraction with EtOAc without (LLE_1) or with acidification (LLE_2) of samples. Values in the same row not sharing lower case superscript letters indicate statistically significant differences among analyzed spiked plasma samples at a confidence level of 95%. Not detected (n.d.).

**Table 2:** Recovery (%R) data of the studied polar phenols after testing ten different sample preparation methods for serum (n = 3).

Analyte	PPT_1	PPT_2	PPT_3	PPT_4	PPT + SPE_1	PPT + SPE_2	PPT + SPE_3	PPT + SPE_4	LLE_1	LLE_2
Isoflavones										
Daidzein	42 ± 10 ^ab^	51 ± 17 ^abcd^	24 ± 4 ^a^	49 ± 4 ^abc^	80 ± 12 ^def^	70 ± 17 ^bcd^	72 ± 7 ^cde^	108 ± 11 ^f^	100 ± 9 ^ef^	66 ± 1 ^bcd^
Formononetin	39 ± 7 ^ab^	47 ± 14 ^abc^	31 ± 2 ^a^	44 ± 3 ^abc^	55 ± 14 ^bc^	107 ± 5 ^e^	66 ± 1 ^cd^	93 ± 6 ^e^	87 ± 6 ^de^	57 ± 2 ^bc^
Genistein	17 ± 24 ^ab^	1 ± 1 ^a^	10 ± 3 ^a^	37 ± 3 ^abc^	80 ± 31 ^d^	92 ± 4 ^d^	58 ± 20 ^bcd^	81 ± 3 ^d^	75 ± 8 ^cd^	60 ± 4 ^cd^
Flavanones										
Hesperetin	10 ± 17 ^a^	n.d.	3 ± 2 ^a^	18 ± 3 ^ab^	62 ± 12 ^cd^	78 ± 8 ^d^	46 ± 17 ^bc^	70 ± 12 ^cd^	68 ± 6 ^cd^	57 ± 3 ^cd^
Naringenin	10 ± 16 ^a^	n.d.	4 ± 2 ^a^	20 ± 3 ^a^	61 ± 11 ^bc^	78 ± 9 ^c^	49 ± 14 ^b^	65 ± 3 ^bc^	73 ± 8 ^bc^	59 ± 2 ^bc^
Flavones										
Apigenin	2 ± 1 ^a^	1 ± 1 ^a^	13 ± 2 ^ab^	32 ± 2 ^bc^	55 ± 14 ^de^	68 ± 6 ^def^	48 ± 12 ^cd^	69 ± 9 ^ef^	82 ± 5 ^f^	53 ± 1 ^cde^
Chrysin	10 ± 13 ^a^	2 ± 3 ^a^	20 ± 1 ^ab^	31 ± 1 ^abc^	65 ± 21 ^de^	71 ± 10 ^de^	51 ± 15 ^cd^	62 ± 11 ^de^	84 ± 3 ^e^	46 ± 4 ^bcd^
Luteolin	8 ± 13 ^a^	1 ± 1 ^a^	8 ± 2 ^a^	30 ± 1 ^ab^	74 ± 24 ^c^	63 ± 7 ^bc^	53 ± 21 ^bc^	64 ± 7 ^bc^	71 ± 14 ^c^	48 ± 6 ^bc^
Flavonols										
Isorhamnetin	5 ± 9 ^ab^	n.d.	18 ± 3 ^abc^	32 ± 1 ^bcd^	74 ± 27 ^e^	57 ± 5 ^de^	46 ± 12 ^cde^	51 ± 8 ^de^	65 ± 4 ^e^	7 ± 5 ^ab^
Kaempferol	12 ± 21 ^a^	n.d.	18 ± 2 ^ab^	34 ± 1 ^abc^	74 ± 24 ^d^	57 ± 5 ^cd^	52 ± 14 ^bcd^	54 ± 8 ^cd^	67 ± 6 ^cd^	8 ± 6 ^a^
Quercetin	4 ± 6 ^a^	n.d.	10 ± 2 ^ab^	29 ± 1 ^abc^	79 ± 28 ^e^	50 ± 5 ^cde^	44 ± 16 ^cd^	37 ± 7 ^bcd^	68 ± 4 ^de^	7 ± 5 ^ab^
Flavan-3-ols										
Catechin	n.d.	n.d.	n.d.	n.d.	64 ± 7 ^bc^	81 ± 3 ^c^	47 ± 20 ^b^	53 ± 4 ^b^	81 ± 9 ^c^	6 ± 4 ^a^
Epicatechin	n.d.	n.d.	n.d.	n.d.	73 ± 27 ^cd^	78 ± 5 ^d^	37 ± 4 ^b^	51 ± 4 ^bc^	69 ± 6 ^cd^	7 ± 2 ^a^
Epicatechin gallate	n.d.	n.d.	n.d.	n.d.	47 ± 12 ^b^	53 ± 8 ^b^	35 ± 14 ^b^	40 ± 1 ^b^	98 ± 19 ^d^	3 ± 3 ^a^
Epigallocatechin	n.d.	n.d.	n.d.	n.d.	39 ± 14 ^b^	60 ± 2 ^bc^	42 ± 18 ^b^	38 ± 9 ^b^	73 ± 12 ^c^	5 ± 4 ^a^
Epigallocatechin gallate	n.d.	n.d.	n.d.	n.d.	37 ± 14 ^bc^	40 ± 7 ^bc^	33 ± 13 ^bc^	29 ± 4 ^b^	55 ± 15 ^c^	n.d.
Procyanidin B2	n.d.	n.d.	n.d.	n.d.	51 ± 30 ^bc^	65 ± 11 ^c^	27 ± 16 ^ab^	37 ± 8 ^bc^	n.d.	n.d.
Flavonol glycosides										
Isoquercetin	11 ± 18 ^ab^	n.d.	n.d.	30 ± 13 ^abc^	70 ± 16 ^cd^	80 ± 14 ^d^	59 ± 24 ^cd^	83 ± 9 ^d^	86 ± 18 ^d^	49 ± 4 ^bcd^
Rutin	n.d.	n.d.	n.d.	29 ± 13 ^abc^	79 ± 34 ^d^	77 ± 11 ^d^	67 ± 27 ^bcd^	89 ± 8 ^d^	71 ± 7 ^cd^	25 ± 4 ^ab^
Stilbenes										
*trans*-Resveratrol	14 ± 24 ^ab^	n.d.	n.d.	n.d.	76 ± 39 ^c^	80 ± 8 ^c^	40 ± 15 ^abc^	47 ± 0 ^bc^	58 ± 5 ^bc^	46 ± 4 ^bc^
E-Viniferin	19 ± 17 ^ab^	4 ± 4 ^a^	n.d.	4 ± 4 ^a^	11 ± 14 ^ab^	24 ± 2 ^ab^	25 ± 13 ^ab^	35 ± 4 ^b^	96 ± 7 ^c^	6 ± 4 ^a^
Piceid	31 ± 17 ^abc^	28 ± 8 ^ab^	n.d.	n.d.	80 ± 17 ^de^	67 ± 18 ^cde^	45 ± 17 ^bcd^	53 ± 4 ^bcde^	87 ± 15 ^e^	26 ± 9 ^ab^
Phenylethanoids										
3-Hydroxytyrosol	27 ± 9 ^a^	30 ± 8 ^ab^	36 ± 2 ^ab^	47 ± 4 ^bc^	76 ± 5 ^d^	80 ± 15 ^d^	74 ± 1 ^d^	115 ± 7 ^e^	63 ± 4 ^cd^	62 ± 4 ^cd^
Oleuropein	45 ± 78 ^ab^	n.d.	n.d.	n.d.	62 ± 2 ^ab^	84 ± 9 ^b^	46 ± 19 ^ab^	52 ± 1 ^ab^	91 ± 9 ^b^	7 ± 6 ^a^
Cinnamic acid and derivatives										
Caffeic acid	16 ± 25 ^abc^	n.d.	10 ± 1 ^ab^	22 ± 5 ^abc^	81 ± 31 ^d^	92 ± 22 ^d^	52 ± 20 ^bcd^	61 ± 2 ^cd^	82 ± 6 ^d^	24 ± 8 ^abc^
*trans*-Cinnamic acid	31 ± 10 ^a^	47 ± 23 ^ab^	28 ± 2 ^a^	39 ± 4 ^a^	72 ± 7 ^ab^	74 ± 24 ^ab^	72 ± 39 ^ab^	98 ± 16 ^b^	61 ± 5 ^ab^	59 ± 2 ^ab^
Chlorogenic acid	44 ± 17 ^bcd^	33 ± 16 ^abc^	n.d.	10 ± 4 ^ab^	71 ± 9 ^d^	73 ± 14 ^d^	67 ± 29 ^cd^	56 ± 6 ^cd^	38 ± 6 ^abcd^	36 ± 5 ^abcd^
*o*-Coumaric acid	36 ± 11 ^ab^	47 ± 17 ^abc^	17 ± 1 ^a^	45 ± 6 ^abc^	73 ± 10 ^cde^	66 ± 18 ^bcd^	65 ± 26 ^bcd^	93 ± 10 ^cd^	106 ± 4 ^d^	68 ± 2 ^bcd^
*p*-Coumaric acid	35 ± 10 ^abc^	44 ± 15 ^bcd^	13 ± 1 ^a^	23 ± 7 ^ab^	69 ± 10 ^de^	65 ± 16 ^de^	51 ± 18 ^bcd^	63 ± 5 ^cde^	88 ± 6 ^e^	67 ± 0 ^de^
Ferulic acid	37 ± 13 ^abc^	44 ± 17 ^abcd^	11 ± 1 ^a^	21 ± 6 ^ab^	76 ± 11 ^de^	64 ± 16 ^cde^	52 ± 20 ^bcde^	60 ± 4 ^cde^	83 ± 3 ^e^	59 ± 4 ^cde^
Neochlorogenic acid	28 ± 13 ^abc^	34 ± 16 ^abc^	n.d.	11 ± 5 ^ab^	83 ± 32 ^de^	95 ± 9 ^e^	46 ± 19 ^bcd^	51 ± 3 ^cd^	n.d.	9 ± 4 ^ab^
Sinapic acid	28 ± 24	28 ± 10	7 ± 1	18 ± 4	81 ± 32	93 ± 16	48 ± 17	57 ± 3	90 ± 8	37 ± 5
Benzoic acid derivatives										
Gallic acid	19 ± 0 ^a^	n.d.	n.d.	41 ± 10 ^abc^	82 ± 32 ^cd^	65 ± 2 ^bcd^	67 ± 0 ^bcd^	98 ± 11 ^d^	34 ± 2 ^ab^	11 ± 6 ^a^
Syringic acid	10 ± 9 ^a^	7 ± 12 ^a^	32 ± 1 ^ab^	46 ± 3 ^abc^	38 ± 0 ^ab^	61 ± 22 ^bc^	70 ± 5 ^bc^	80 ± 9 ^c^	68 ± 11 ^bc^	17 ± 3 ^a^
Vanillic acid	41 ± 8 ^ab^	54 ± 22 ^ab^	33 ± 1 ^a^	55 ± 7 ^ab^	63 ± 2 ^ab^	59 ± 21 ^ab^	74 ± 15 ^b^	68 ± 15 ^ab^	72 ± 7 ^b^	76 ± 7 ^b^

Data are presented as mean ± SD. Treatments as follows: Protein precipitation with ACN (PPT_1) or 10% MeOH (PPT_2) or ac. ACN (PPT_3) or ac. 10% MeOH (PPT_4), protein precipitation with ACN followed by SPE (PPT + SPE_1) or 10% MeOH followed by SPE (PPT + SPE_2) or ac. ACN followed by SPE (PPT + SPE _3) or ac. 10% MeOH followed by SPE (PPT + SPE _4), liquid–liquid extraction with EtOAc without (LLE_1) or with acidification (LLE_2) of samples. Values in the same row not sharing lower case superscript letters indicate statistically significant differences among analyzed spiked plasma samples at a confidence level of 95%. Not detected (n.d.).

**Table 3:** Recovery (%R) of the analytes after treating plasma and serum samples with enzymatic hydrolysis conditions in the absence (−) and presence (+) of β-glucuronidase/sulfatase (n = 3).

Analyte	Plasma		Serum	
	(−)	(+)	(−)	(+)
	Isoflavones			
Daidzein	80 ± 7 ^a^	69 ± 6 ^b^	73 ± 7 ^a^	73 ± 4 ^a^
Formononetin	57 ± 7 ^a^	27 ± 2 ^b^	66 ± 6 ^a^	24 ± 2 ^b^
Genistein	53 ± 2 ^a^	51 ± 6 ^a^	55 ± 9 ^a^	56 ± 4 ^a^
	Flavanones			
Hesperetin	70 ± 4 ^a^	59 ± 6 ^b^	78 ± 8 ^a^	68 ± 4 ^b^
Naringenin	46 ± 6 ^a^	22 ± 3 ^b^	46 ± 2 ^a^	35 ± 2 ^b^
	Flavones			
Apigenin	76 ± 2 ^a^	47 ± 4 ^b^	68 ± 2 ^a^	56 ± 1 ^b^
Chrysin	85 ± 4 ^a^	56 ± 2 ^b^	63 ± 1 ^a^	35 ± 1 ^b^
Luteolin	34 ± 4 ^a^	49 ± 8 ^b^	41 ± 9 ^a^	58 ± 6 ^b^
	Flavonols			
Isorhamnetin	62 ± 9 ^a^	13 ± 2 ^b^	66 ± 5 ^a^	33 ± 1 ^b^
Kaempferol	51 ± 4 ^a^	23 ± 3 ^b^	48 ± 6 ^a^	20 ± 2 ^b^
Quercetin	42 ± 3 ^a^	2.2 ± 1 ^b^	49 ± 1 ^a^	6 ± 1 ^b^
	Flavan-3-ols			
Catechin	67 ± 1 ^a^	50 ± 5 ^b^	59 ± 3 ^a^	55 ± 2 ^a^
Epicatechin	63 ± 4 ^a^	57 ± 5 ^a^	59 ± 8 ^a^	60 ± 2 ^a^
Epicatechin gallate	106 ± 5 ^a^	42 ± 8 ^b^	81 ± 6 ^a^	31 ± 0.3 ^b^
Epigallocatechin	27 ± 2 ^a^	11 ± 5 ^b^	15 ± 2 ^a^	22 ± 3 ^a^
Epigallocatechin gallate	62 ± 5 ^a^	26 ± 1 ^b^	53 ± 3 ^a^	20 ± 1 ^b^
Procyanidin B2	6 ± 0 ^a^	7 ± 0 ^b^	7 ± 1 ^a^	5 ± 0 ^b^
	Flavonol glycosides			
Isoquercetin	59 ± 2 ^a^	n.d.	52 ± 3 ^a^	n.d.
Rutin	27 ± 2 ^a^	n.d.	42 ± 4 ^a^	n.d.
	Stilbenes			
*trans*-Resveratrol	67 ± 4 ^a^	54 ± 6 ^b^	79 ± 11 ^a^	57 ± 3 ^b^
E-Viniferin	33 ± 3 ^a^	22 ± 2 ^b^	28 ± 4 ^a^	33 ± 2 ^a^
Piceid	67 ± 3 ^a^	2 ± 1 ^b^	70 ± 4 ^a^	n.d.
	Phenylethanoids			
3-Hydroxytyrosol	74 ± 5 ^b^	58 ± 5 ^a^	80 ± 5 ^a^	71 ± 3 ^a^
Oleuropein	66 ± 1 ^a^	n.d.	55 ± 4 ^a^	n.d.
	Cinnamic acid and derivatives			
Caffeic acid	29 ± 4 ^a^	30 ± 1.2 ^a^	35 ± 4 ^a^	26 ± 1 ^a^
*trans*-Cinnamic acid	32 ± 4 ^a^	27 ± 1 ^a^	35 ± 3 ^a^	31 ± 2 ^a^
Chlorogenic acid	4 ± 1 ^a^	1 ± 1 ^a^	3 ± 1 ^a^	n.d.
*o*-Coumaric acid	61 ± 6 ^a^	123 ± 8 ^b^	62 ± 5 ^a^	109 ± 5 ^b^
*p*-Coumaric acid	66 ± 3 ^a^	50 ± 1 ^a^	52 ± 3 ^a^	66 ± 1 ^ab^
Ferulic acid	97 ± 6 ^a^	93 ± 5 ^a^	83 ± 7 ^a^	78 ± 2 ^a^
Neochlorogenic acid	6 ± 1 ^a^	2 ± 1 ^a^	3 ± 1 ^a^	1 ± 1 ^a^
Sinapic acid	57 ± 2 ^a^	16 ± 1 ^b^	49 ± 5 ^a^	10 ± 2 ^b^
	Benzoic acid derivatives			
Gallic acid	38 ± 2 ^a^	24 ± 5 ^b^	66 ± 2 ^a^	68 ± 0 ^a^
Syringic acid	n.d.	n.d.	n.d.	n.d.
Vanillic acid	55 ± 4 ^a^	62 ± 5 ^ab^	44 ± 2 ^a^	43 ± 1 ^a^

Data are presented as mean ± SD. Values in the same row not sharing lower case superscript letters indicate statistically significant differences among analyzed spiked plasma and serum samples in each case at a confidence level of 95%. Not detected (n.d.).

**Table 4:** Polar phenolic compounds (ng/mL) detected in rat serum samples (n = 6 per group).

Analyte	C	Cenz	CC	CCenz
Isoflavones				
Daidzein	<LOQ	5.9 ± 2.21 ^c^	4.35 ± 1.62 ^ad^	16.89 ± 6.59 ^d^
Formononetin	0.57 ± 0.08 ^ac^	34.11 ± 7.39 ^c^	1.85 ± 0.54 ^ad^	85.45 ± 31.19 ^d^
Genistein	<LOQ	17.83 ± 6.12 ^bc^	<LOQ	40.4 ± 16.28 ^bd^
Flavanones				
Hesperetin	<LOQ	2.44 ± 0.79 ^c^	<LOQ	9.8 ± 3.82 ^d^
Naringenin	<LOQ	3.77 ± 0.83 ^c^	<LOQ	8.61 ± 2.97 ^d^
Flavones				
Apigenin	<LOQ	45.75 ± 15.08 ^bc^	1.53 ± 0.36 ^ad^	181.66 ± 48.95 ^bd^
Chrysin	n.d.	n.d.	<LOQ	2.54 ± 1.06 ^bd^
Luteolin	0.71 ± 0.44 ^c^	6.99 ± 3.63 ^b^	1.07 ± 0.62 ^cd^	113.74 ± 66.08 ^bd^
Flavonols				
Isorhamnetin	<LOQ	<LOQ	<LOQ	5.93 ± 2.22 ^bd^
Kaempferol	<LOQ	4.43 ± 3.34 ^c^	<LOQ	7.52 ± 2.3 ^d^
Quercetin	<LOQ	<LOQ	<LOQ	8.54 ± 4.83 ^d^
Cinnamic acid and derivatives				
*trans*-Cinnamic acid	76.8 ± 32.92 ^c^	188.37 ± 89.04 ^c^	99.35 ± 41.79 ^d^	255.57 ± 116.85 ^d^
Benzoic acid derivatives				
Vanillic acid	47.92 ± 17.64 ^ac^	705.75 ± 44.9 ^c^	438.92 ± 162.4 ^ad^	897.81 ± 173.96 ^d^
Syringic acid	3.45 ± 1.20 ^c^	17.65 ± 8.33 ^c^	28.5 ± 8.29 ^d^	43.2 ± 10.85 ^d^

Data are presented as mean ± SD. Values in the same row sharing lower case superscript letters indicate statistically significant differences among pairs of mean values. Samples C vs. Cenz and CC vs. Cenz were compared with paired T-tests, whereas samples C vs. CC and Cenz vs. CCenz were compared with independent T-tests (^a^ *p* < 0.05 (C-CC), ^b^ *p* < 0.05 (Cenz-CCenz), ^c^ *p* < 0.05 (C-Cenz) and ^d^ *p* < 0.05 (CC-CCenz)). Groups and serum samples’ treatment: control diet samples (C), enzymatically treated control diet samples (Cenz), Corinthian Currant supplemented diet samples (CC) and enzymatically treated Corinthian Currant supplemented diet samples (CCenz). Not detected (n.d.). Lower than limit of quantitation (<LOQ).

## Data Availability

Not applicable.

## Supplementary Materials

The following supporting information can be downloaded at: https://www.mdpi.com/article/10.3390/biom12121838/s1.
