# Supplementary material for: Polar Phenol Detection in Plasma and Serum: Insights on Sample Pre-Treatment for LC/MS Analysis and Application on the Serum of Corinthian Currant-Fed Rats
Source: Biomolecules. 2022 Dec 8;12(12):1838. doi: 10.3390/biom12121838 (PMC9775334; doi:10.3390/biom12121838)
Supplement: Supplementary file 1 [file biomolecules-12-01838-s001.zip › biomolecules-1990476-supplementary.pdf]

Supplementary Material

Table S1. Chromatographic and mass spectral data acquired in (-)-HESI for each analyte and the internal standard (IS).

Analyte	RT (min)	Chemical Formula	Elemental Composition	Theoretical Mass [M-H]-	Measured Mass [M-H]-	Mass error (ppm)	Fragment Ions (AIF) 35 eV (NCE)
**Isoflavones**							
Daidzein	10.62	$C_{15}H_{10}O_4$	$C_{15}H_9O_4^-$	253.0506	253.0507	-0.40	n.d.
Formononetin	12.72	$C_{16}H_{12}O_4$	$C_{16}H_{11}O_4^-$	267.0663	267.0667	-1.50	n.d.
Genistein	12.04	$C_{15}H_{10}O_5$	$C_{15}H_9O_5^-$	269.0455	269.0459	-1.49	241.0507/ 225.0553/ 213.0553/ 169.065/ 133.0281/
**Flavanones**							
Hesperetin	12.25	$C_{16}H_{14}O_6$	$C_{16}H_{13}O_6^-$	301.0718	301.0720	-0.66	n.d.
Naringenin	12.07	$C_{15}H_{12}O_5$	$C_{15}H_{11}O_5^-$	271.0612	271.0616	-1.48	93.0329/ 119.0487/ 151.0025/ 119.0487/ 177.0184/ 271.0616
**Flavones**							
Apigenin	11.89	$C_{15}H_{10}O_5$	$C_{15}H_9O_5^-$	269.0455	269.0457	-0.74	225.0553/ 227.0346/ 213.0553/ 151.0026/ 149.0233/ 117.033
Chrysin	13.33	$C_{15}H_{10}O_4$	$C_{15}H_9O_4^-$	253.0506	253.0507	-0.40	107.0122/ 89.0128
Luteolin	10.89	$C_{15}H_{10}O_6$	$C_{15}H_9O_6^-$	285.0405	285.0406	-0.35	217.0503/ 199.0394/ 151.0025
**Flavonols**							
Isorhamnetin	12.18	$C_{16}H_{12}O_7$	$C_{16}H_{11}O_7^-$	315.0510	315.0514	-1.27	300.0278
Kaempferol	12.11	$C_{15}H_{10}O_6$	$C_{15}H_9O_6^-$	285.0405	285.0406	-0.35	243.0299/ 185.0600/ 169.0649/ 151.0025/ 123.0073/ 107.0122
Quercetin	11.08	$C_{15}H_{10}O_7$	$C_{15}H_9O_7^-$	301.0354	301.0355	-0.33	151.0025/ 178.9977/ 107.0122/ 229.0504/ 273.0413
**Flavan-3-ols**							

Catechin	8.27	$C_{15}H_{14}O_6$	$C_{15}H_{16}O_6^-$	289.0718	289.0720	-0.69	245.0817/ 205.0499/ 179.0344/ 165.0182/ 137.020 / 109.0279
Epicatechin	8.58	$C_{15}H_{14}O_6$	$C_{15}H_{13}O_6^-$	289.0718	289.0720	-0.69	245.0821/ 205.0503/ 179.0341/ / 165.0182/ 137.031/ 109.0279
Epicatechin gallate	9.18	$C_{22}H_{18}O_{10}$	$C_{22}H_{17}O_{10}^-$	441.0827	441.0831	-0.91	n.d.
Epigallocatechin	7.89	$C_{15}H_{14}O_7$	$C_{15}H_{13}O_7^-$	305.0667	305.0668	-0.33	n.d.
Epigallocatechin gallate	8.67	$C_{22}H_{18}O_{11}$	$C_{22}H_{17}O_{11}^-$	457.0776	457.0783	-1.53	n.d.
**Flavonol glycosides**							
Isoquercetin	8.97	$C_{21}H_{20}O_{12}$	$C_{21}H_{19}O_{12}^-$	463.0882	463.0891	-1.94	301.0350/ 300.0279
Rutin	8.77	$C_{27}H_{30}O_{16}$	$C_{27}H_{29}O_{16}^-$	609.1461	609.1469	-1.31	301.0361/ 300.0278/ 343.0461/ 271.0252
**Stilbenes**							
trans-Resveratrol	10.56	$C_{14}H_{12}O_3$	$C_{14}H_{11}O_3^-$	227.0714	227.0711	1.32	185.0599/ 143.0490
E-Viniferin	11.68	$C_{28}H_{22}O_6$	$C_{28}H_{21}O_6^-$	453.1344	453.1351	-1.54	n.d.
Piceid	9.03	$C_{20}H_{22}O_8$	$C_{20}H_{21}O_8^-$	389.1242	389.1251	-2.31	227.0711
**Phenylethanoids**							
3-Hydroxytyrosol	7.59	$C_8H_{10}O_3$	$C_8H_9O_3^-$	153.0546	153.0544	1.31	123.0436
Oleuropein	9.53	$C_{25}H_{32}O_{13}$	$C_{25}H_{31}O_{13}^-$	539.1770	539.1779	1.67	377.1239, 275.0923, 307.0821
**Cinnamic acid and derivatives**							
trans-Cinnamic acid	12.00	$C_9H_8O_2$	$C_9H_7O_2^-$	147.0441	147.0438	2.04	n.d.
Caffeic acid	8.75	$C_9H_8O_4$	$C_9H_7O_4^-$	179.0339	179.0341	-1.12	135.0438
Chlorogenic acid	8.08	$C_{16}H_{18}O_9$	$C_{16}H_{17}O_9^-$	353.0878	353.0882	-1.13	n.d.
o-Coumaric acid	10.22	$C_9H_8O_3$	$C_9H_7O_3^-$	163.0390	163.0387	1.84	119.0487
p-Coumaric acid	9.39	$C_9H_8O_3$	$C_9H_7O_3^-$	163.0390	163.0388	1.23	191.0554/ 179.0341
Ferulic acid	9.53	$C_{10}H_{10}O_4$	$C_{10}H_9O_4^-$	193.0495	193.0499	-2.07	178.0263/ 149.0596/ 134.0359
Neochlorogenic acid	7.56	$C_{16}H_{18}O_9$	$C_{16}H_{17}O_9^-$	353.0878	353.0883	-1.42	n.d.
Sinapic acid	9.41	$C_{11}H_{12}O_5$	$C_{11}H_{11}O_5^-$	223.0612	223.0608	1.79	n.d.

**Benzoic acid derivatives**							
Gallic acid	4.68	$C_7H_6O_5$	$C_7H_5O_5^-$	169.0131	169.0132	-0.59	125.0229/ 97.0278/ 81.0329
Syringic acid	8.81	$C_9H_{10}O_5$	$C_9H_9O_5^-$	197.0444	197.0447	-1.52	182.0212/ 23.0079/ 121.0280/ 95.0122
Vanillic acid	9.58	$C_8H_8O_4$	$C_8H_7O_4^-$	167.0339	167.0337	1.20	152.0103/ 123.0436/ 108.0201
**Internal Standard**							
Quercetin-d3 (IS)	11.02	$C_{15}H_7D_3O_7$	$C_{15}H_6D_3O_7^-$	304.0531	304.0543	3.95	n.d.

Figures S1a-S9b: Example extracted ion chromatograms (XICs) of selected polar phenolics for each pre-treatment method tested for plasma and serum samples.

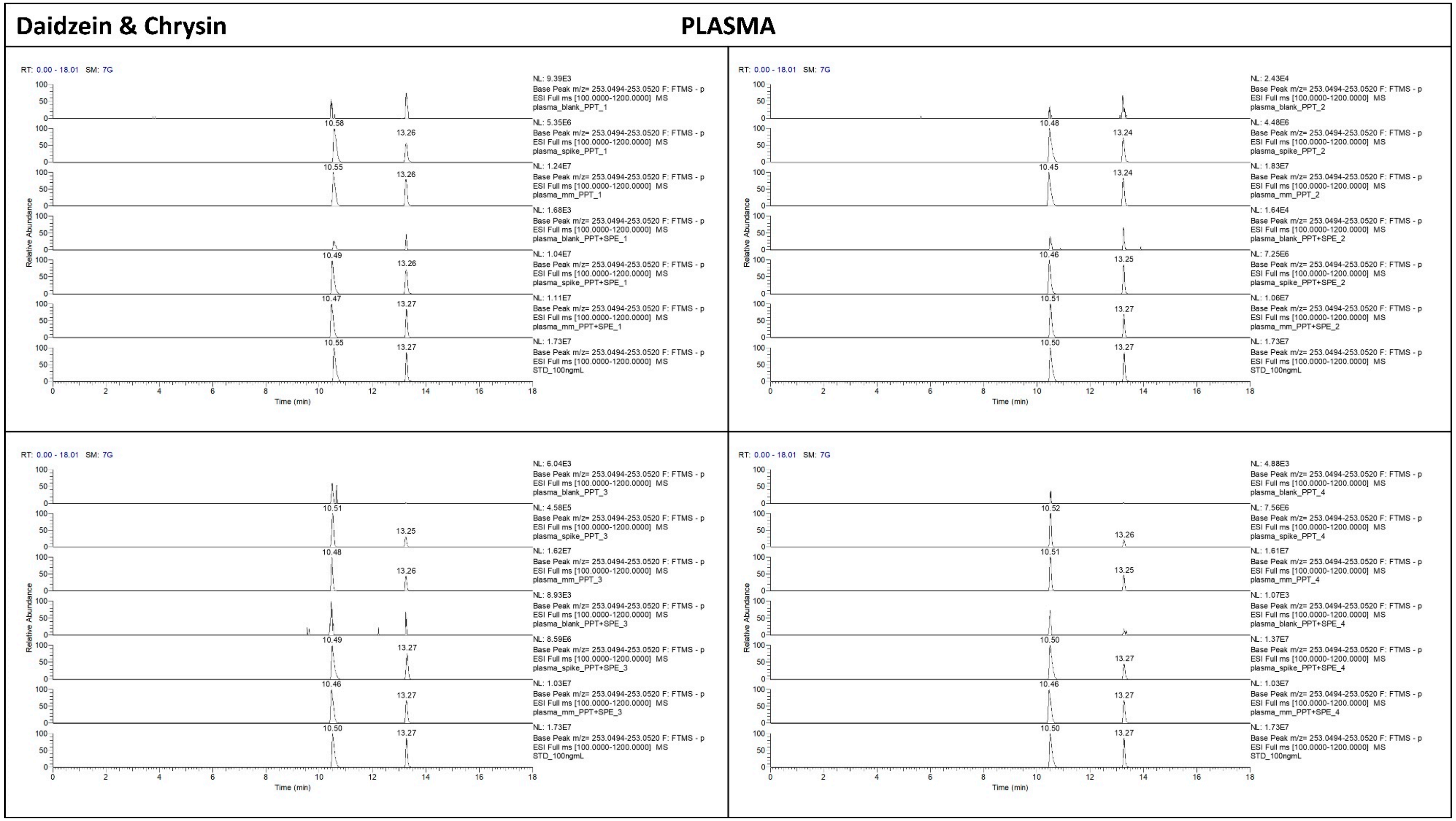

Figure S1a. Extracted ion chromatograms (XICs) of daidzein (10.50 min) and chrysin (13.27 min) for each pre-treatment method tested for plasma samples.

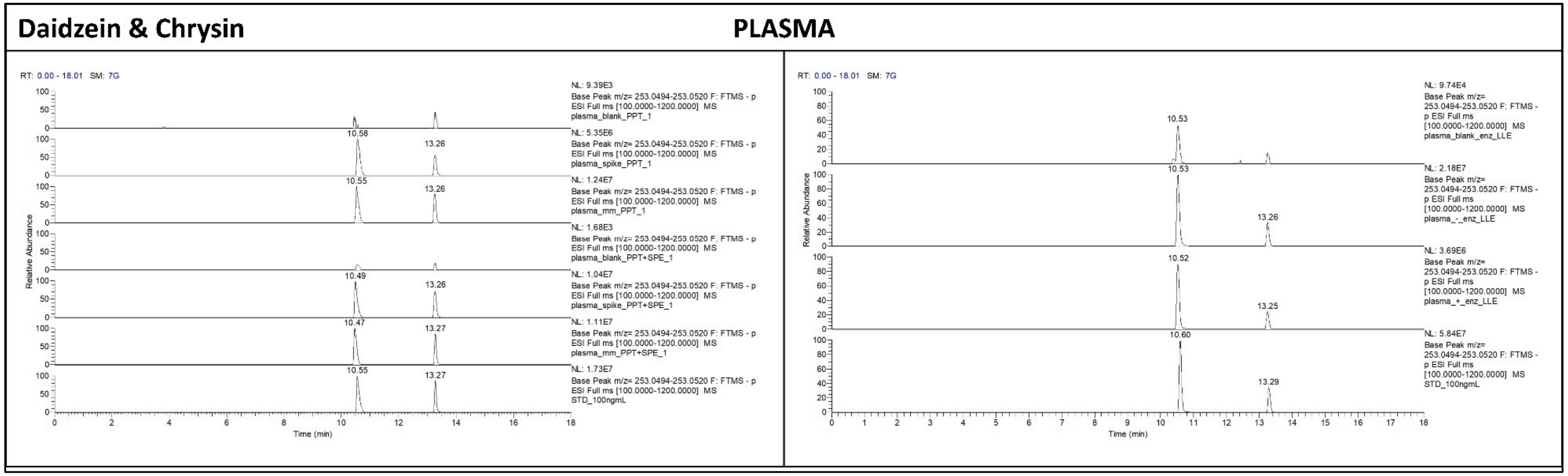

Figure S1a. (continued)

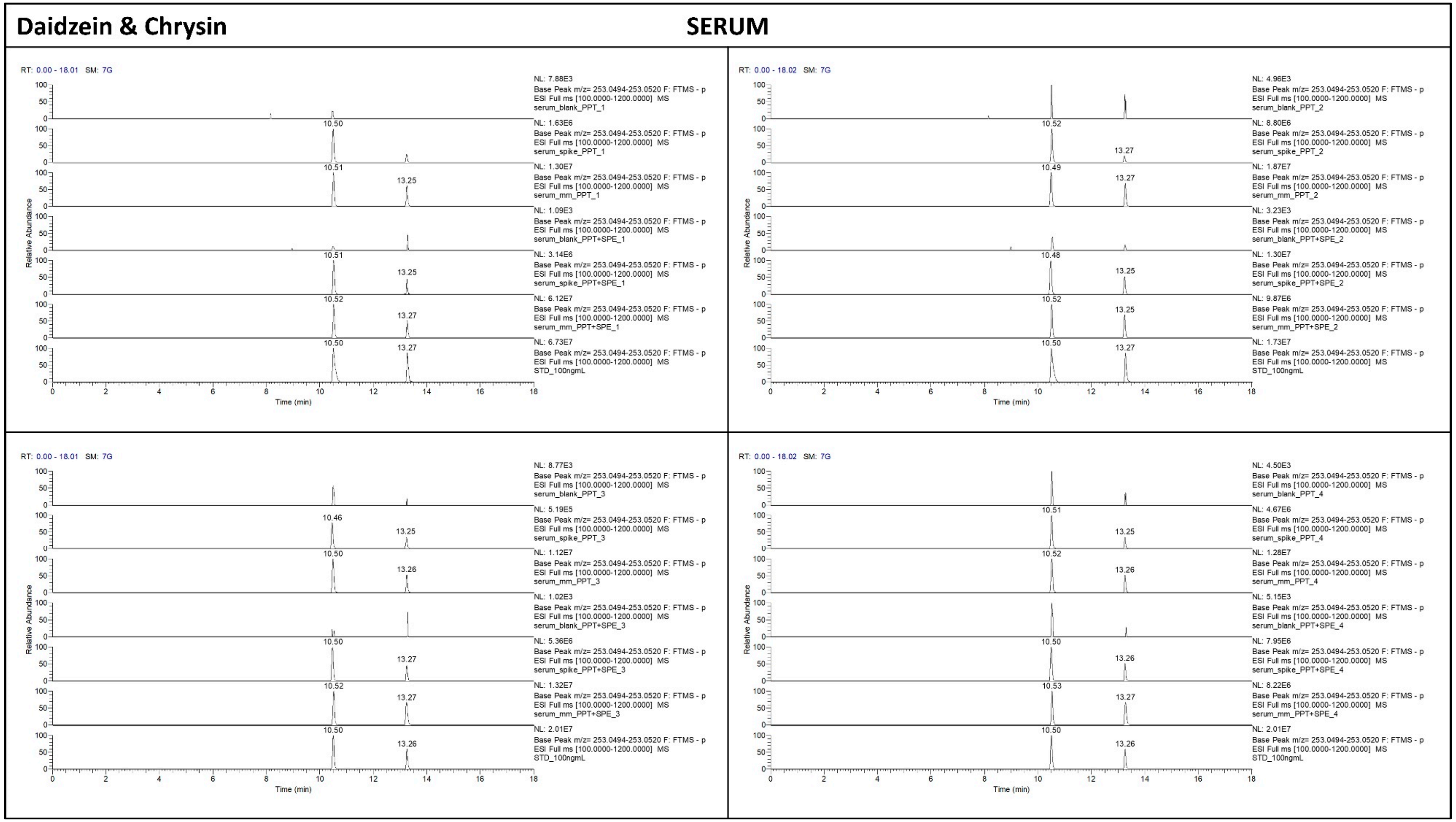

Figure S1b. Extracted ion chromatograms (XICs) of daidzein (10.50 min) and chrysin (13.27 min) for each pre-treatment method tested for serum samples.

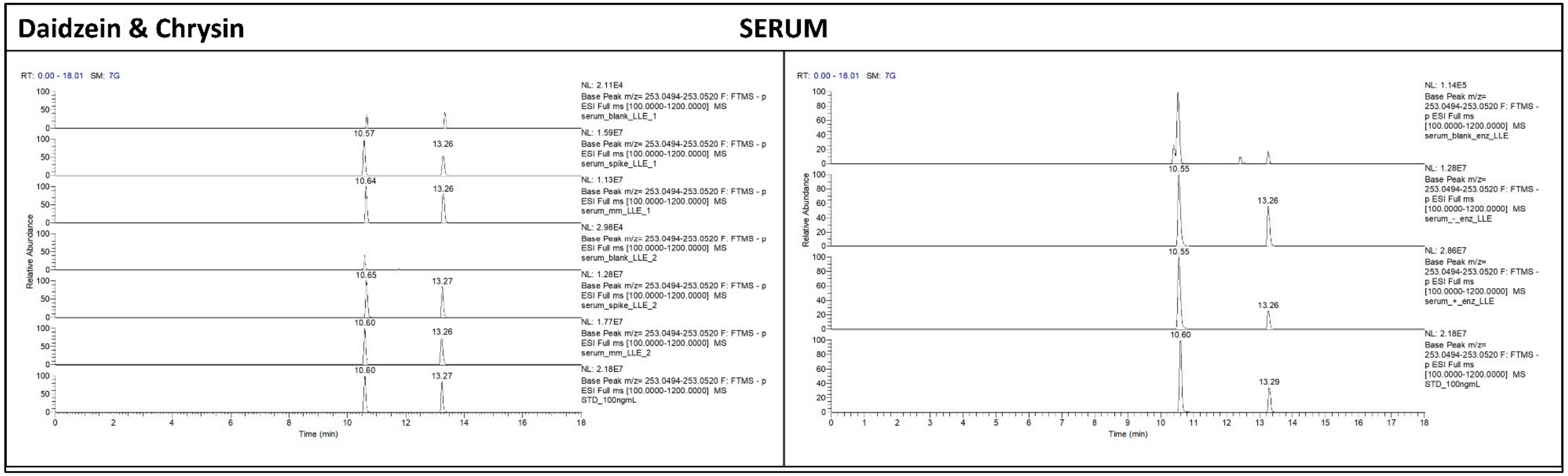

Figure S1b. (continued)

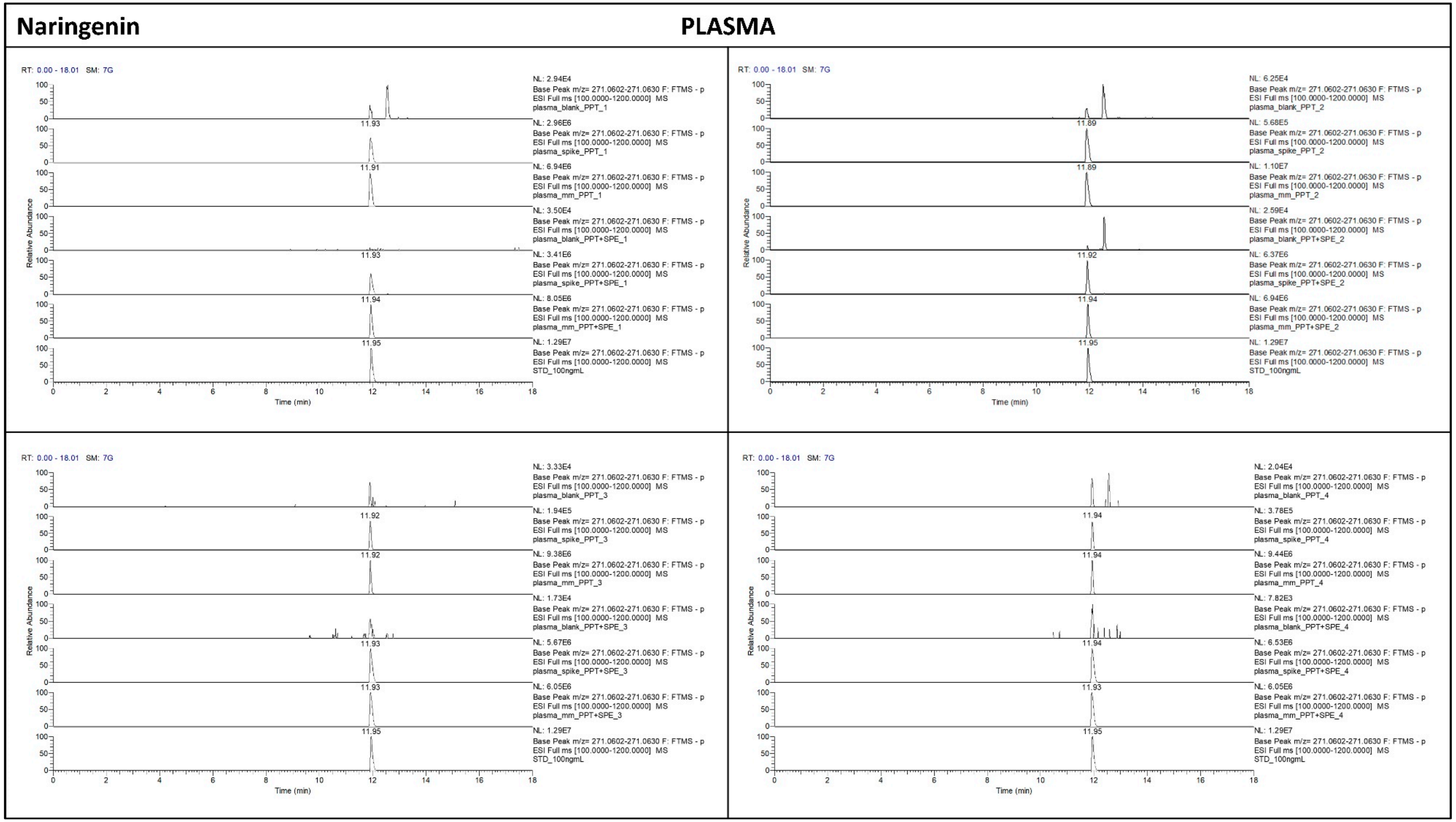

Figure S2a. Extracted ion chromatograms (XICs) of naringenin (11.95 min) for each pre-treatment method tested for plasma samples.

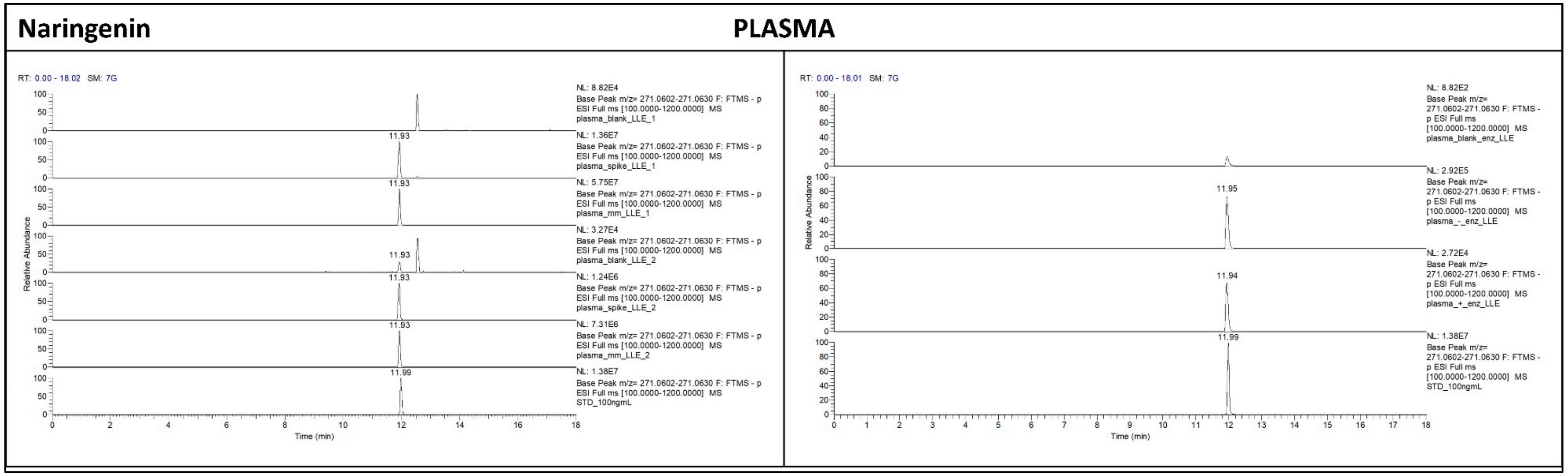

Figure S2a. (continued)

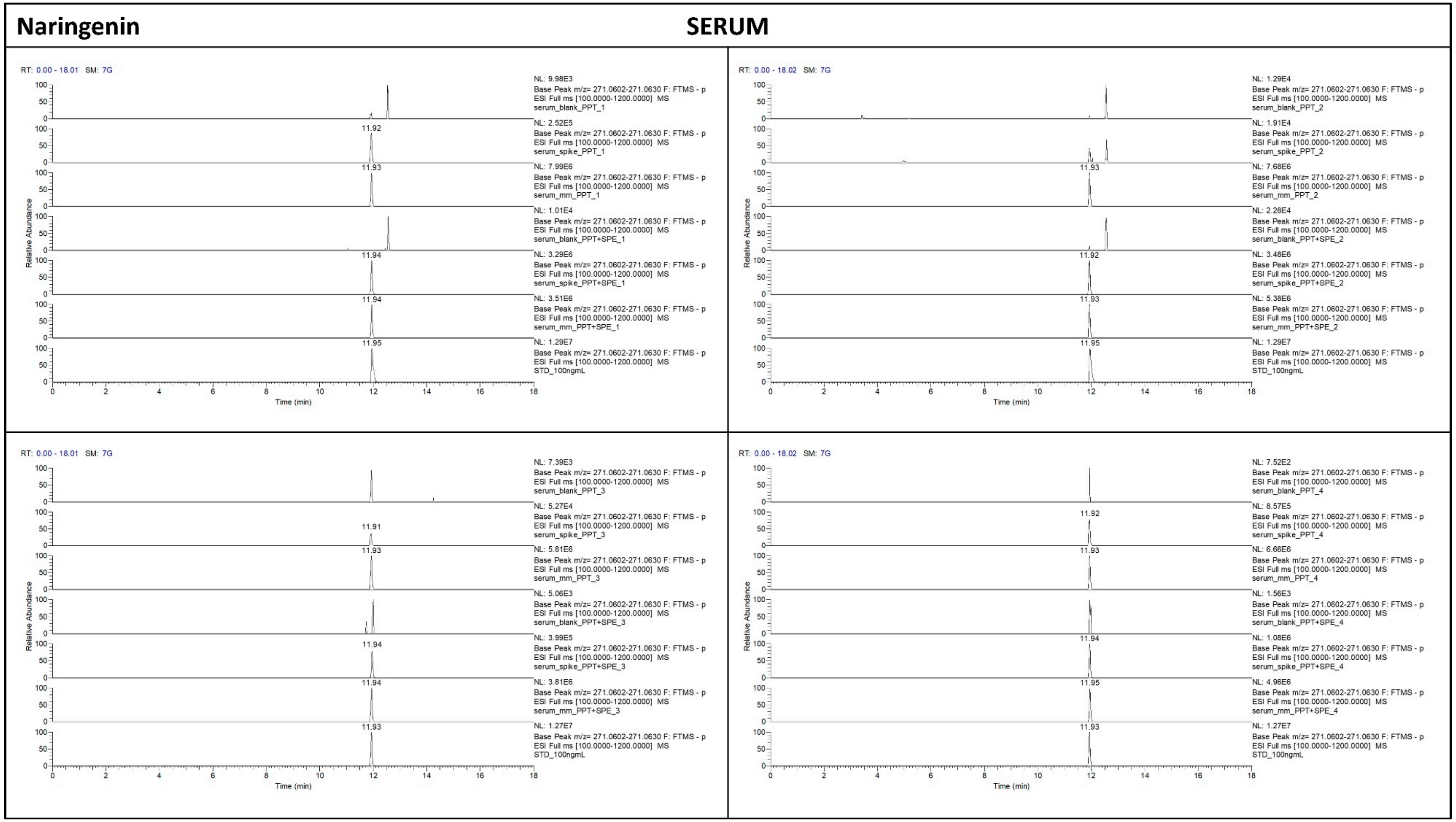

Figure S2b. Extracted ion chromatograms (XICs) of naringenin (11.95 min) for each pre-treatment method tested for serum samples.

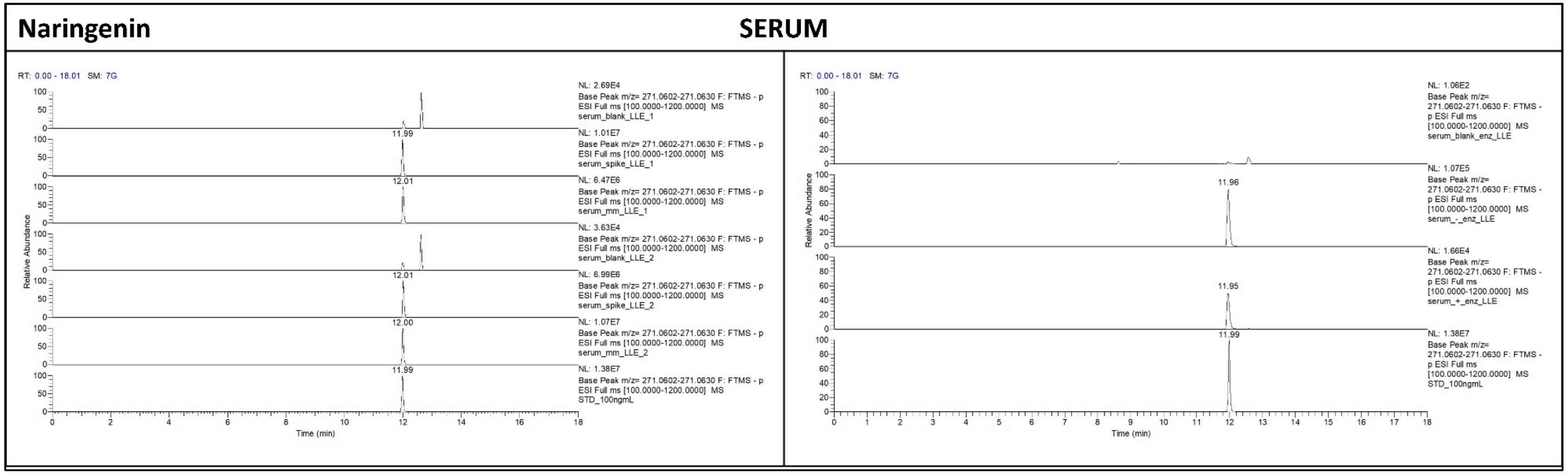

Figure S2b. (continued)

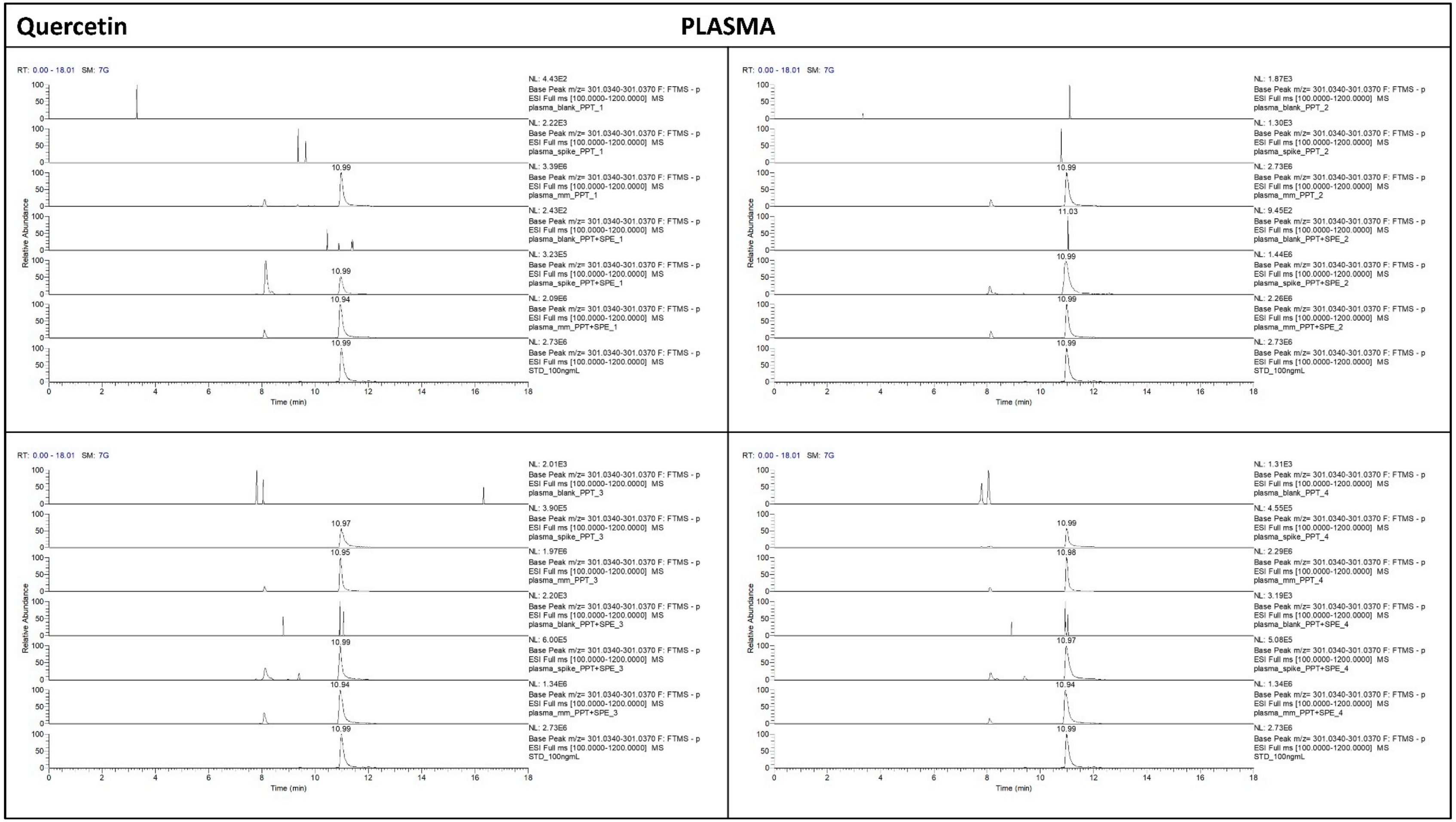

Figure S3a. Extracted ion chromatograms (XICs) of quercetin (10.99 min) for each pre-treatment method tested for plasma samples.

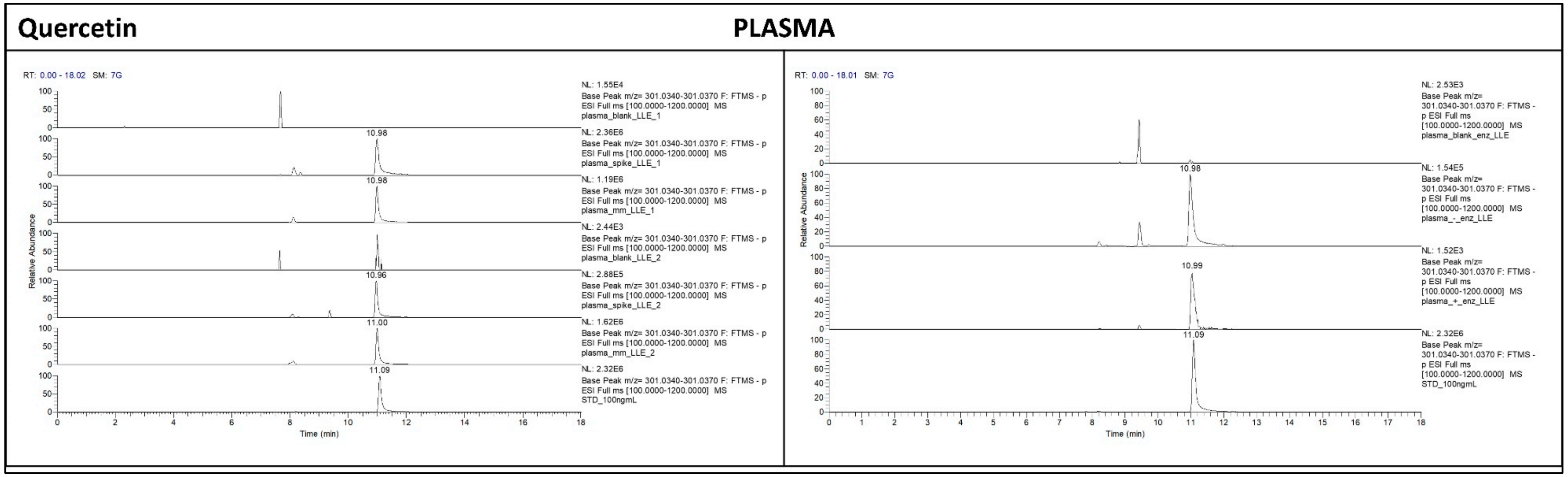

Figure S3a. (continued)

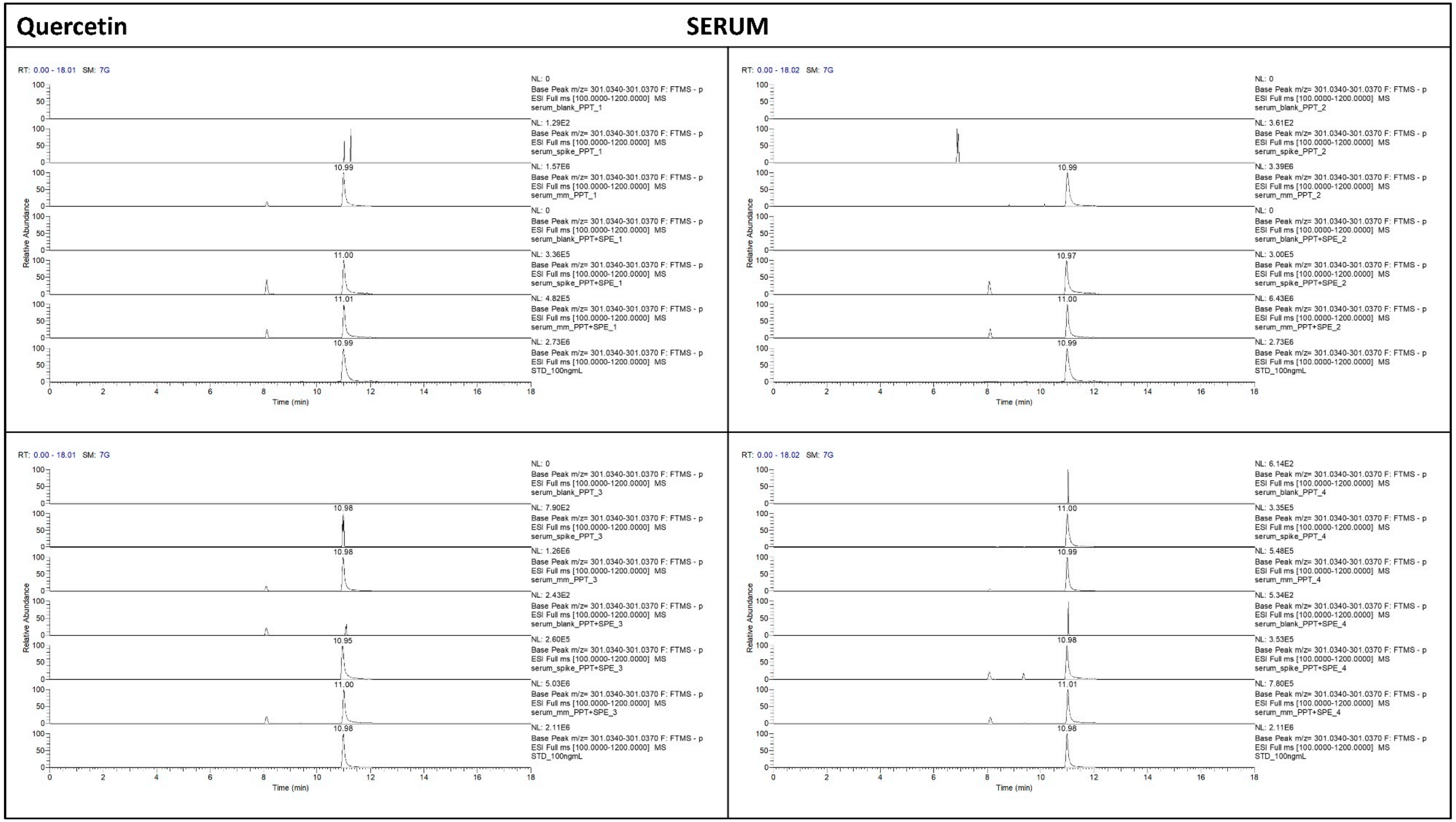

Figure S3b. Extracted ion chromatograms (XICs) of quercetin (10.99 min) for each pre-treatment method tested for serum samples.

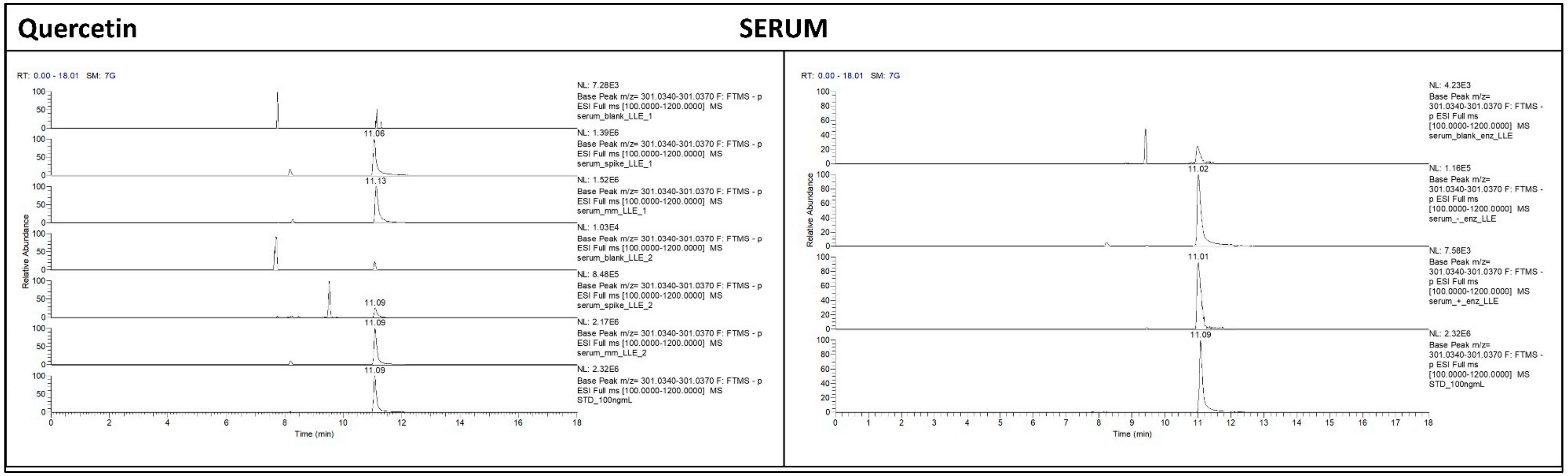

Figure S3b. (continued)

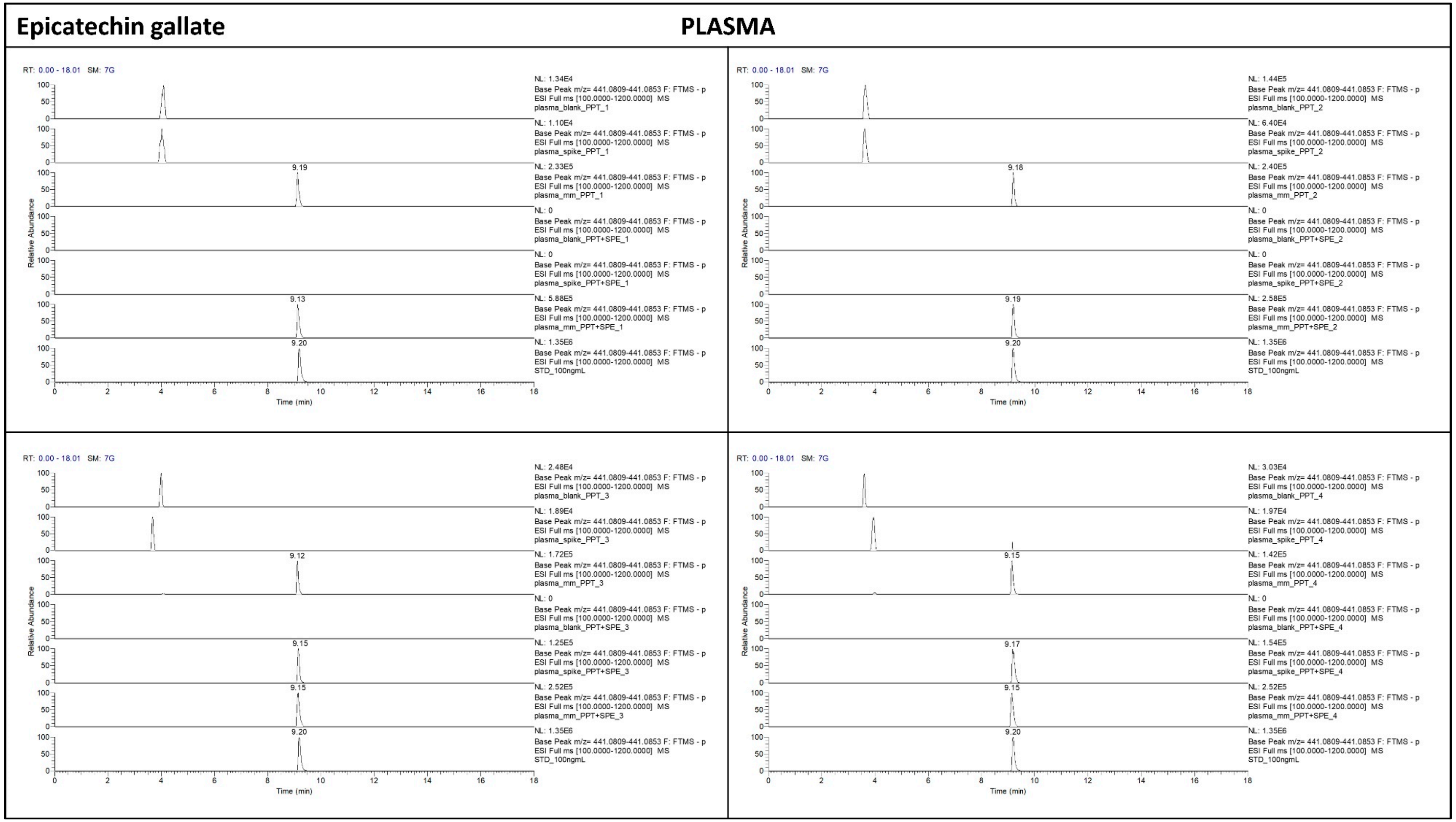

Figure S4a. Extracted ion chromatograms (XICs) of epicatechin gallate (9.20 min) for each pre-treatment method tested for plasma samples.

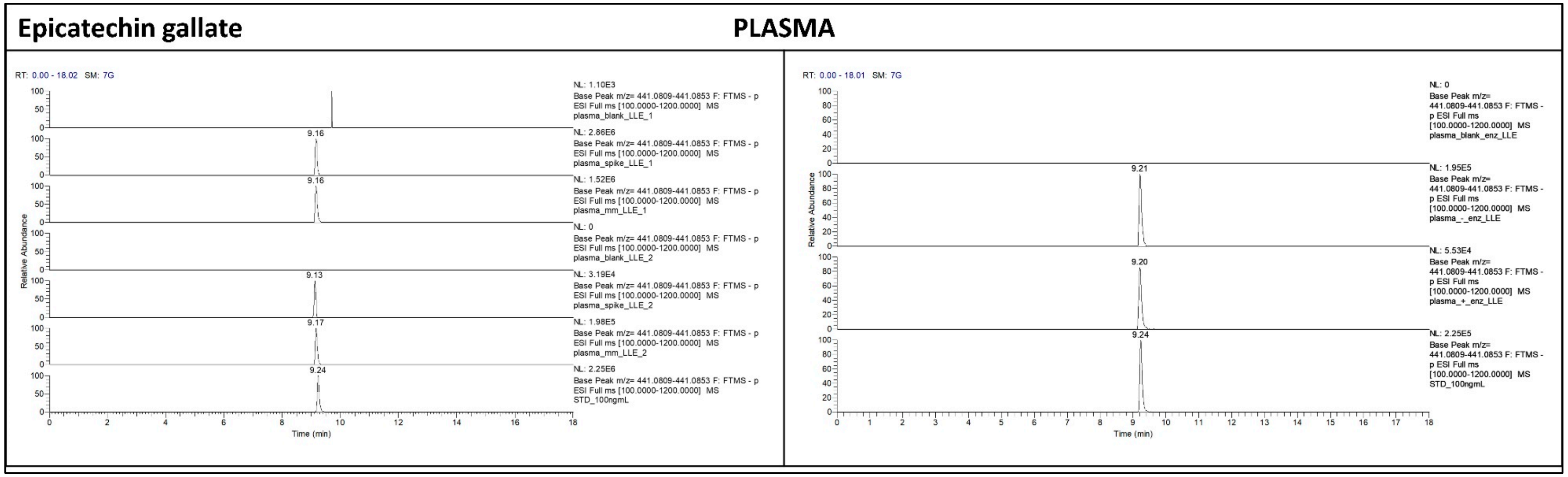

Figure S4a. (continued)

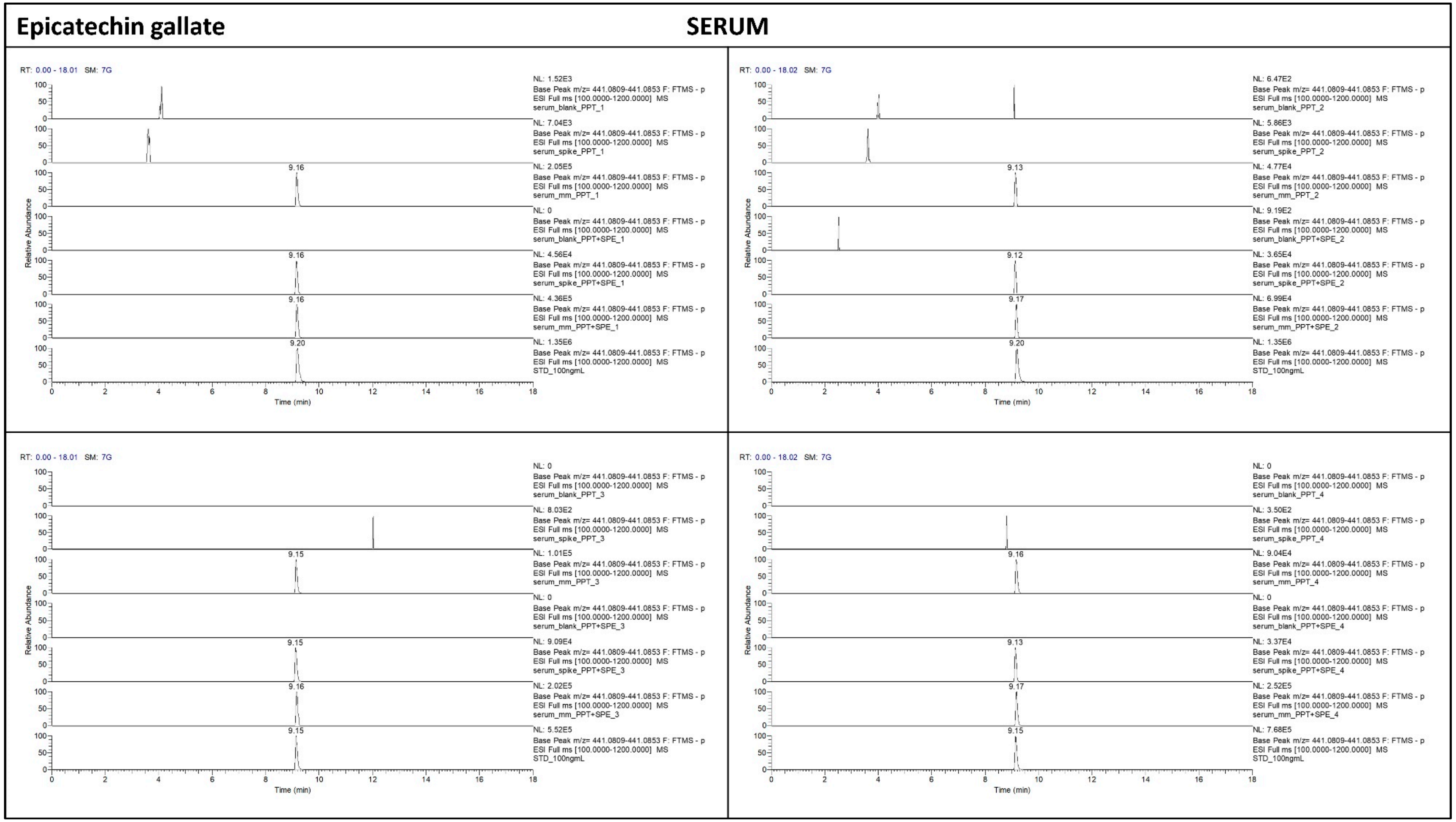

Figure S4b. Extracted ion chromatograms (XICs) of epicatechin gallate (9.20 min) for each pre-treatment method tested for serum samples.

Epicatechin gallate **SERUM**

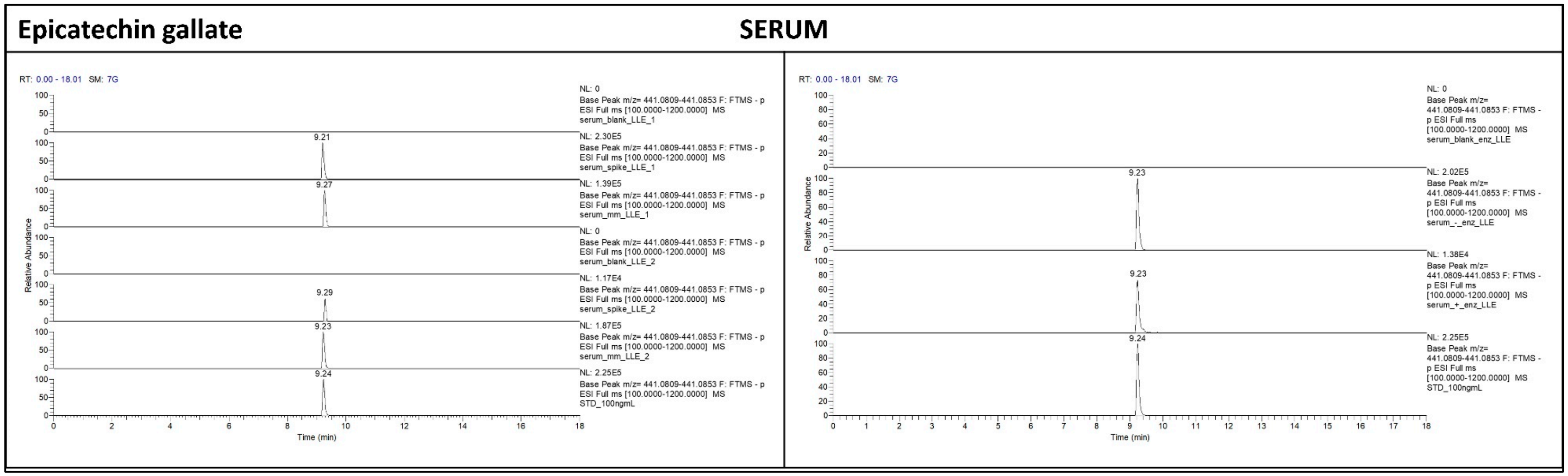

Figure S4b. (continued)

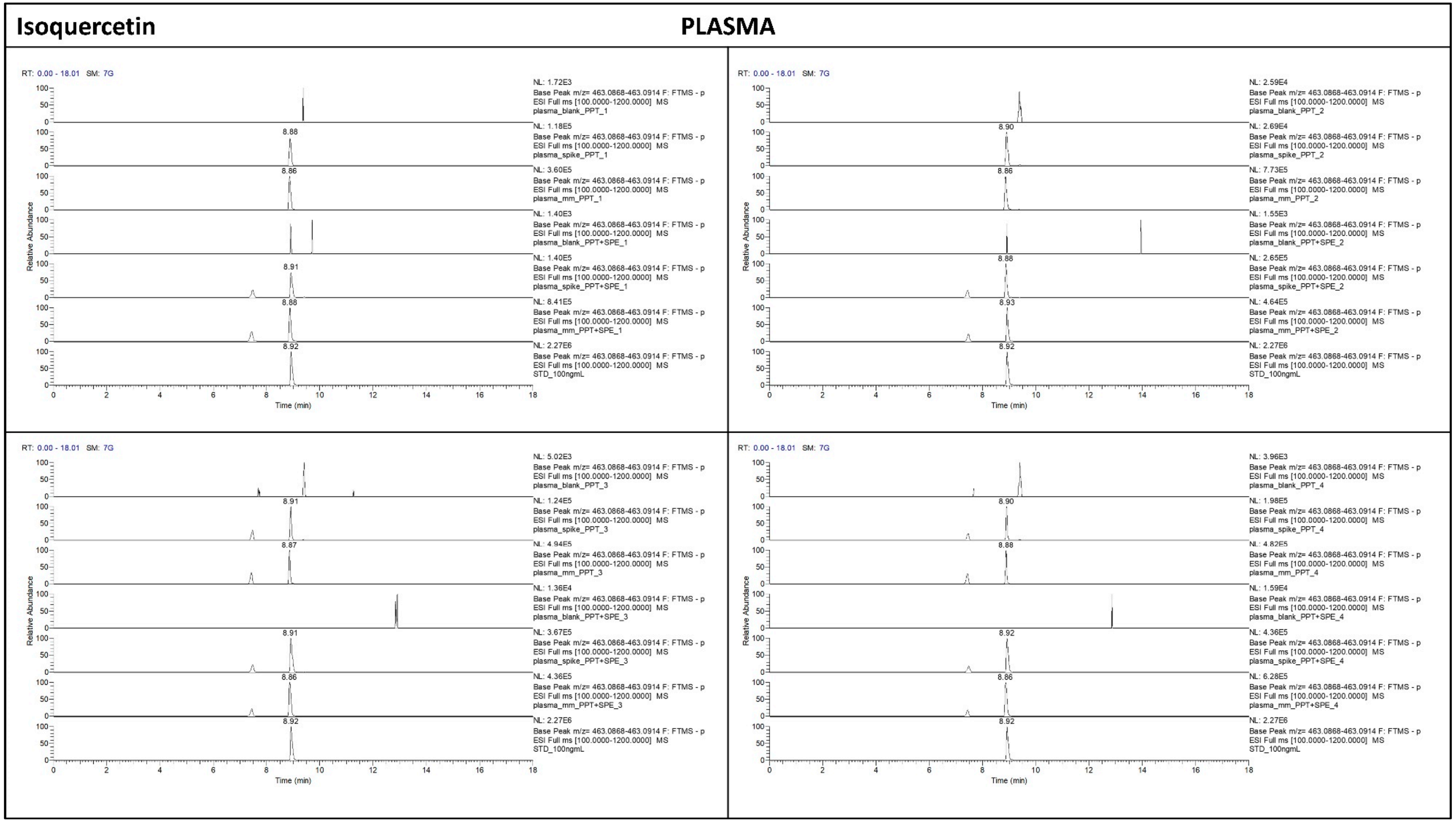

Figure S5a. Extracted ion chromatograms (XICs) of isoquercetin (8.92 min) for each pre-treatment method tested for plasma samples.

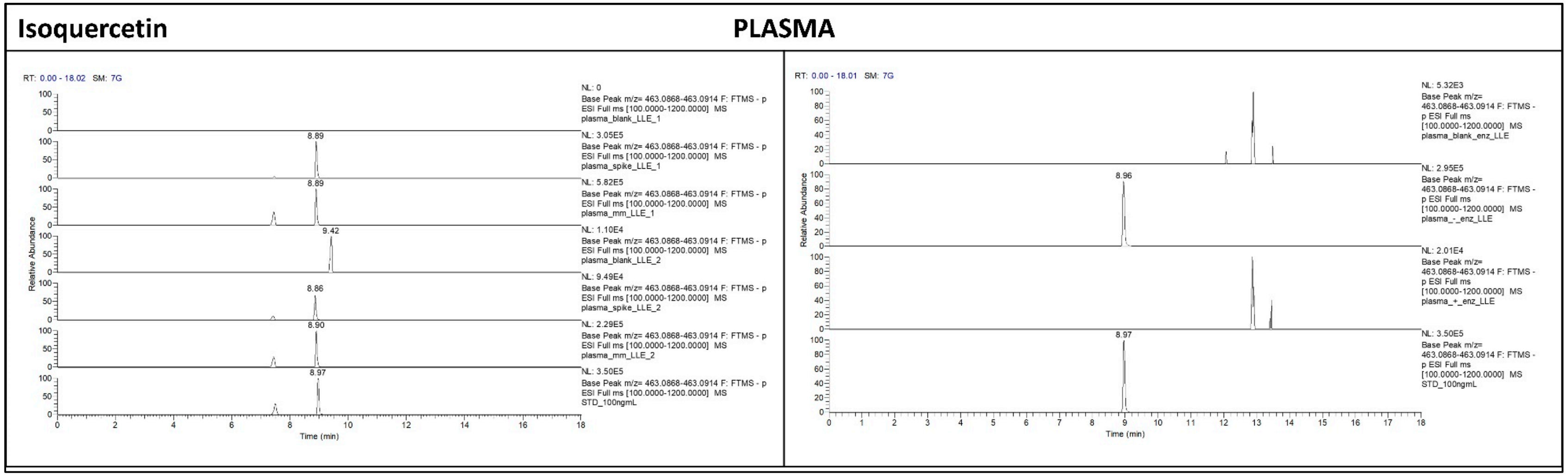

Figure S5a. (continued)

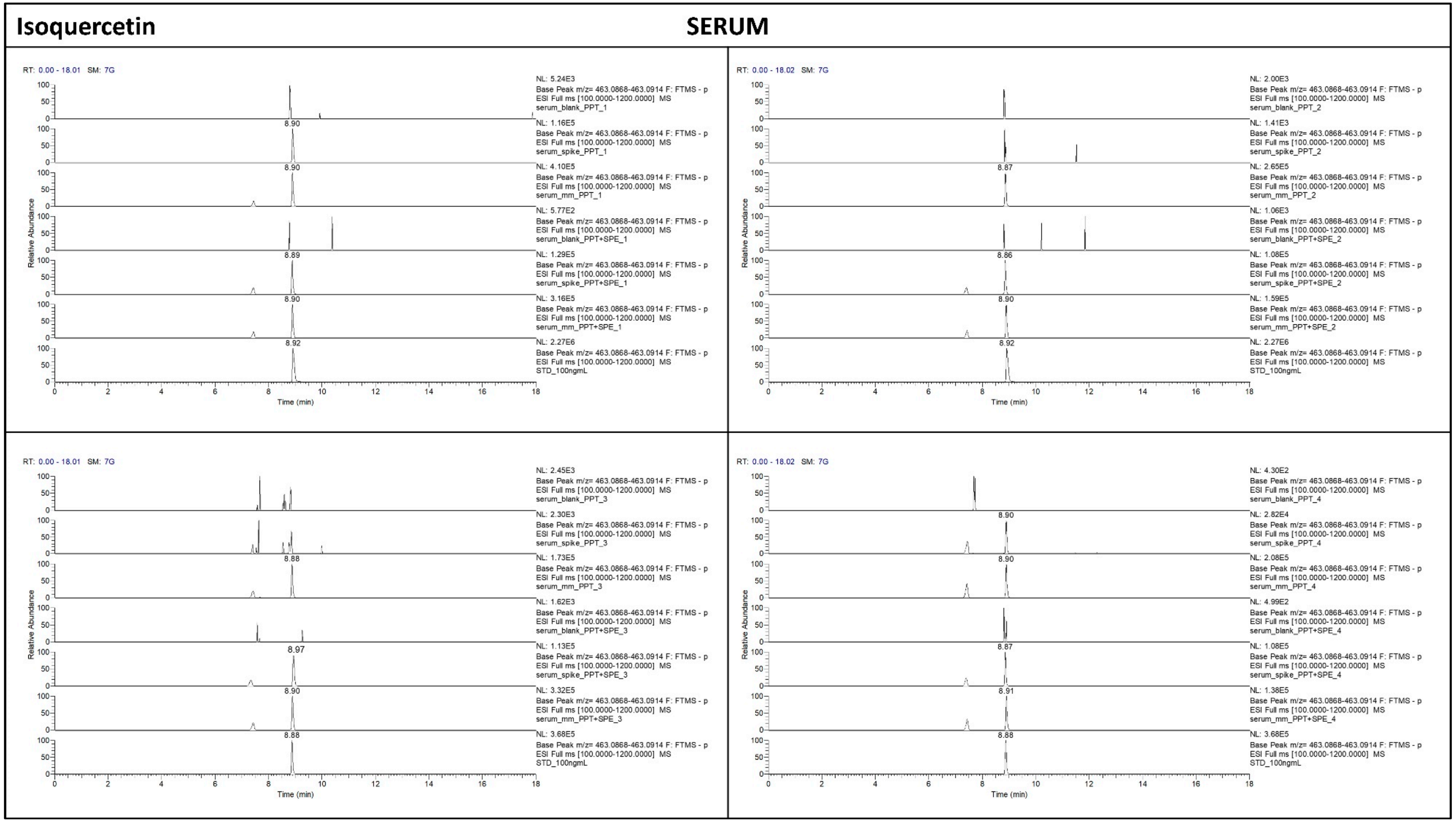

Figure S5b. Extracted ion chromatograms (XICs) of isoquercetin (8.92 min) for each pre-treatment method tested for serum samples.

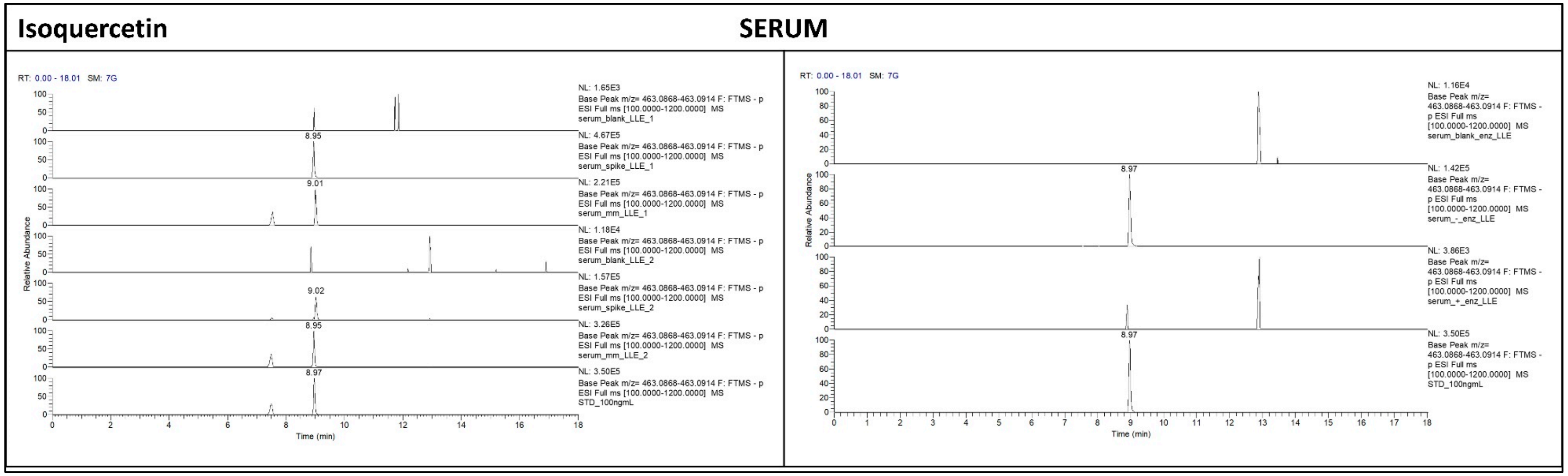

Figure S5b. (continued)

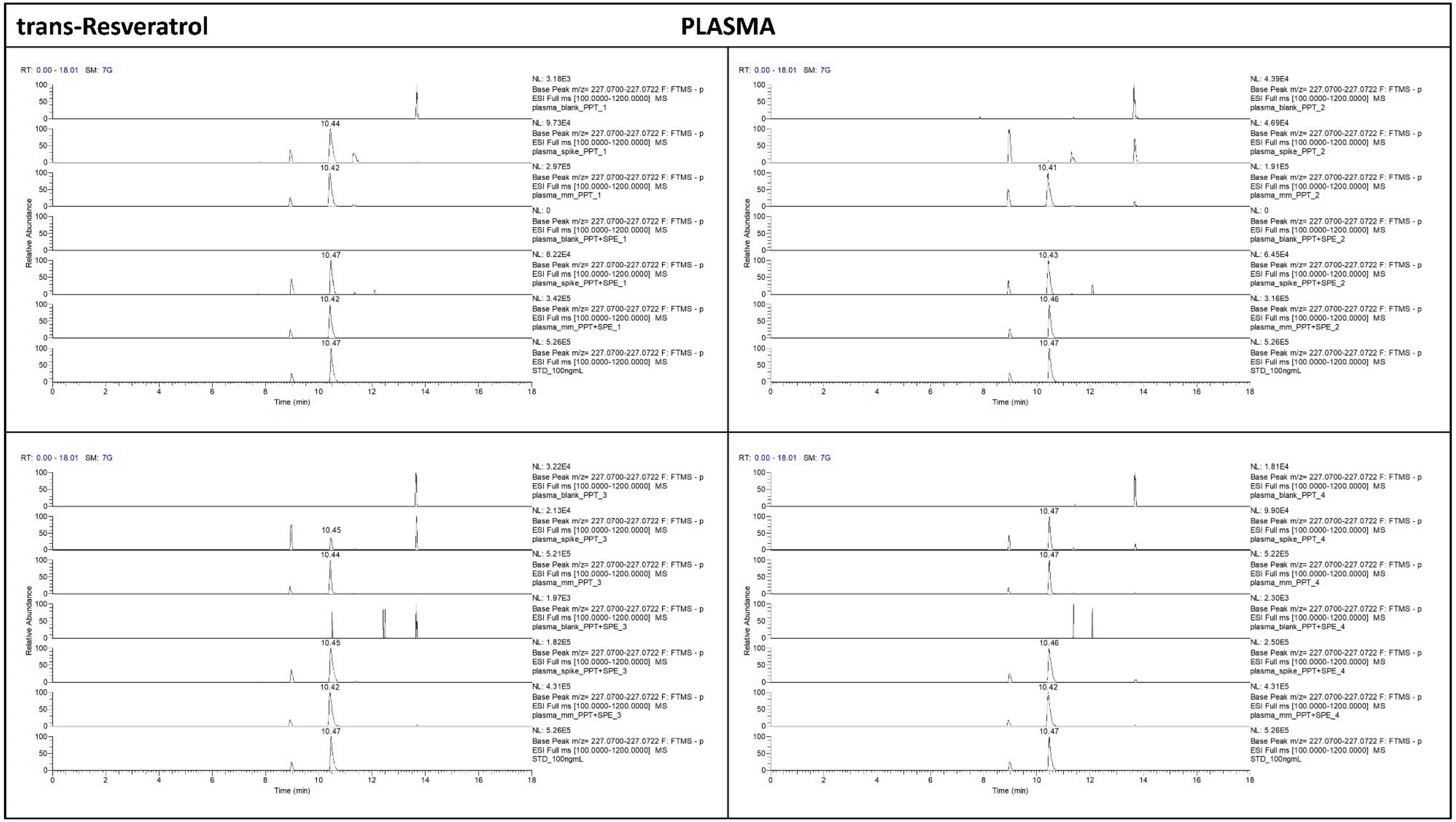

Figure S6a. Extracted ion chromatograms (XICs) of trans-resveratrol (10.47 min) for each pre-treatment method tested for plasma samples.

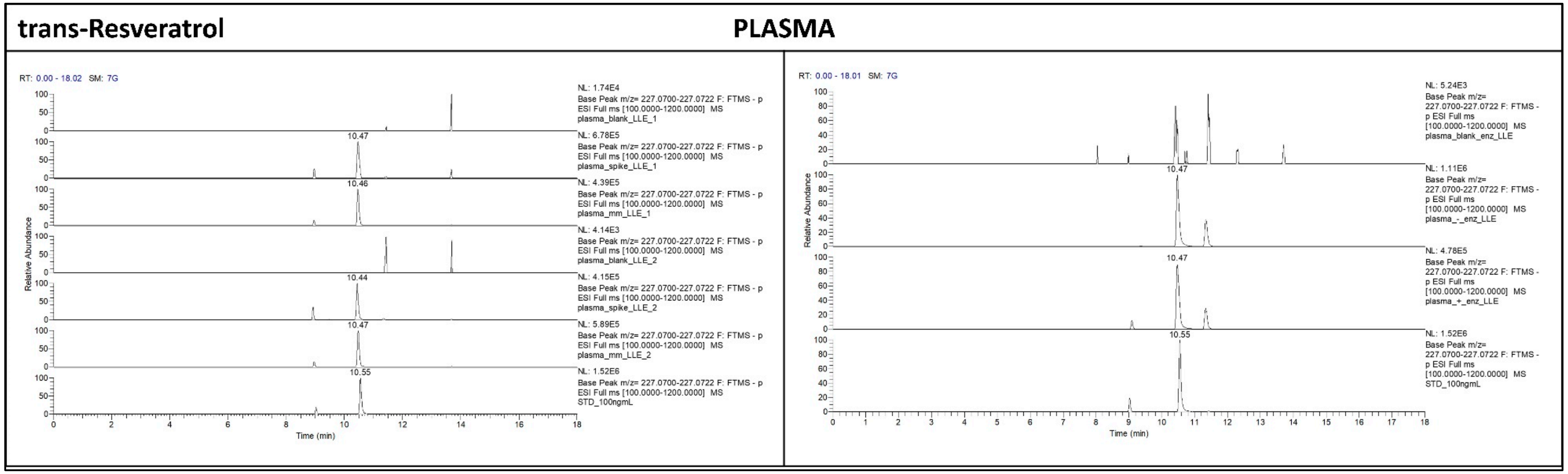

Figure S6a. (continued)

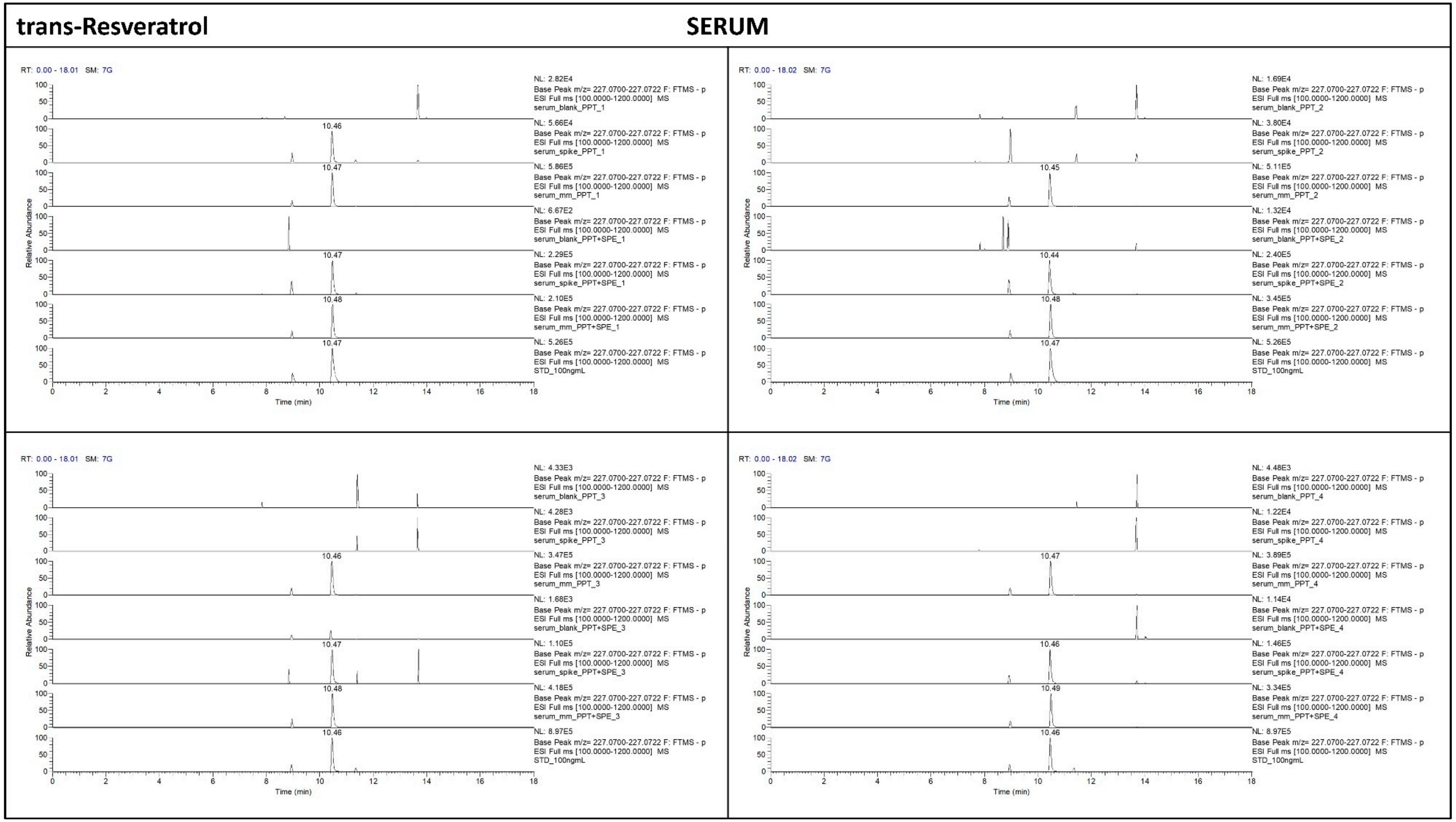

Figure S6b. Extracted ion chromatograms (XICs) of trans-resveratrol (10.47 min) for each pre-treatment method tested for serum samples.

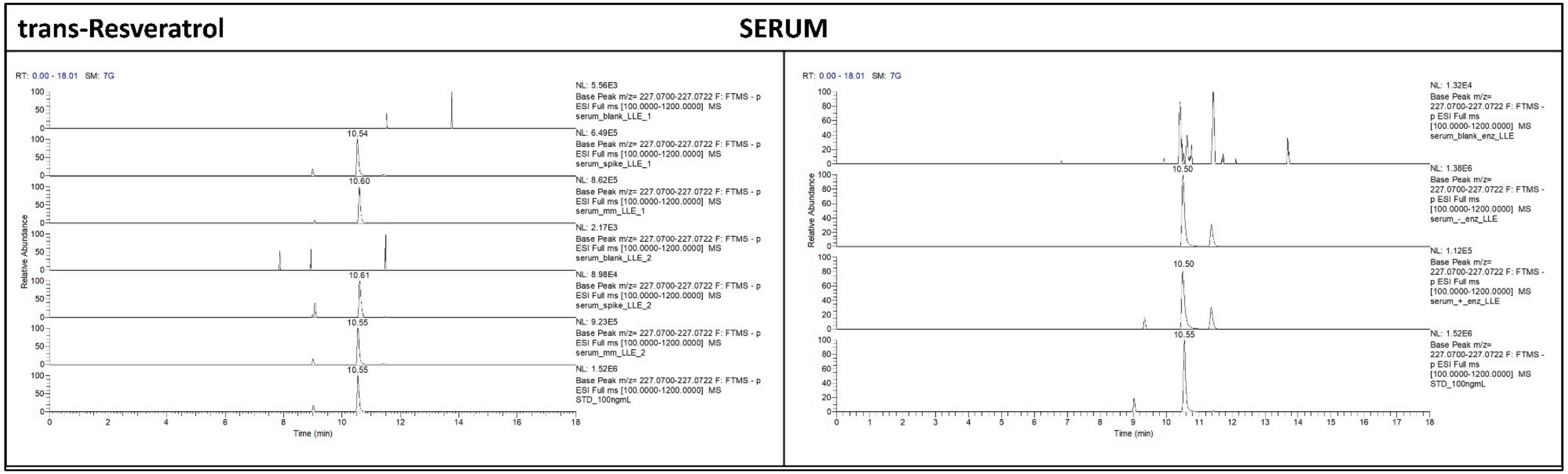

Figure S6b. (continued)

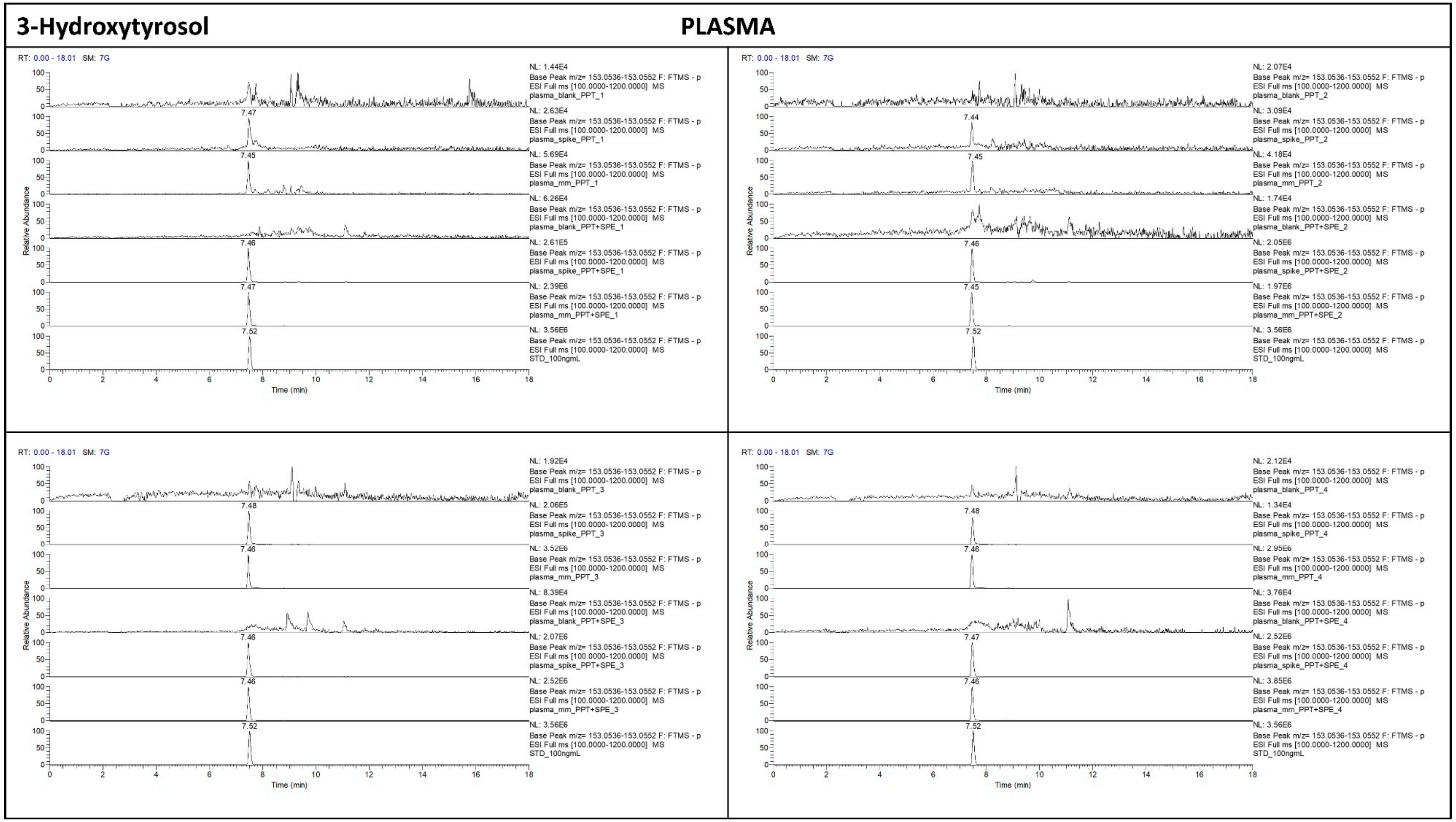

Figure S7a. Extracted ion chromatograms (XICs) of 3-hydroxytyrosol (7.50 min) for each pre-treatment method tested for plasma samples.

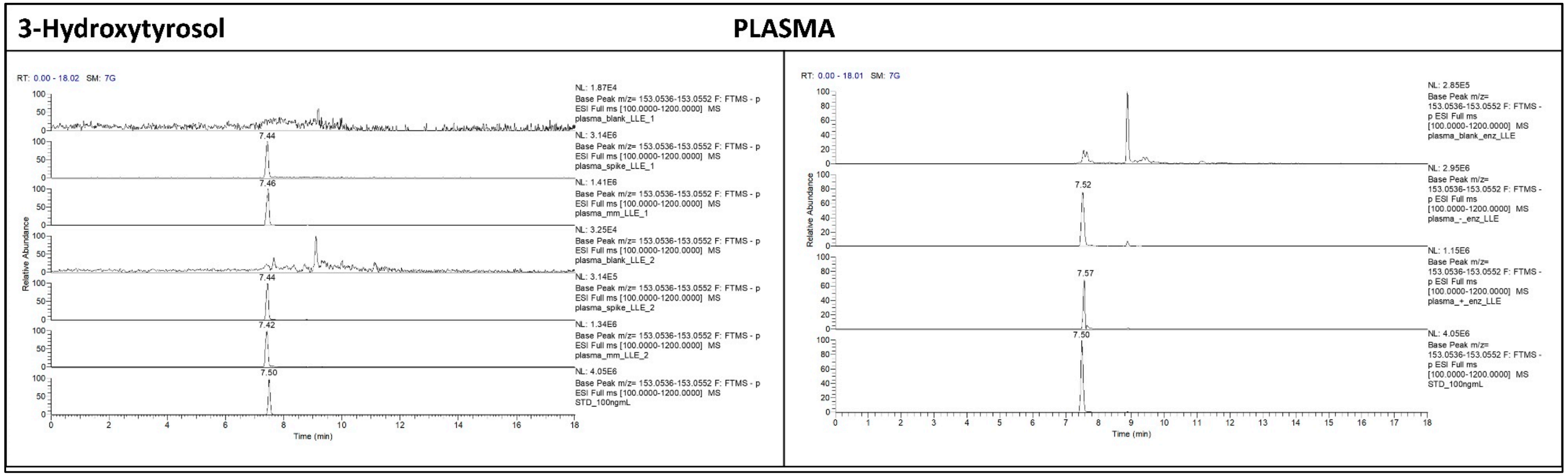

Figure S7a. (continued)

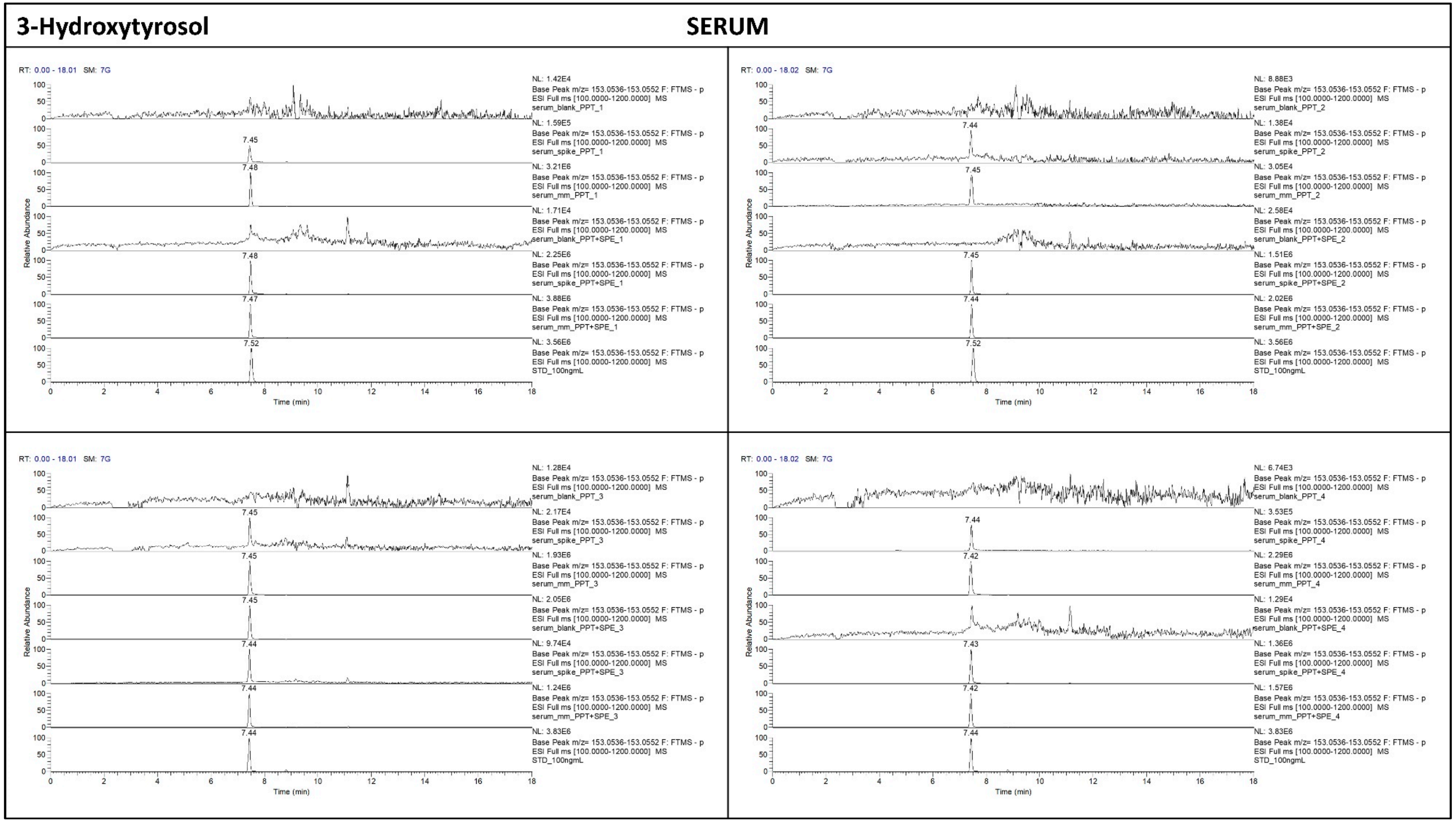

Figure S7b. Extracted ion chromatograms (XICs) of 3-hydroxytyrosol (7.50 min) for each pre-treatment method tested for serum samples.

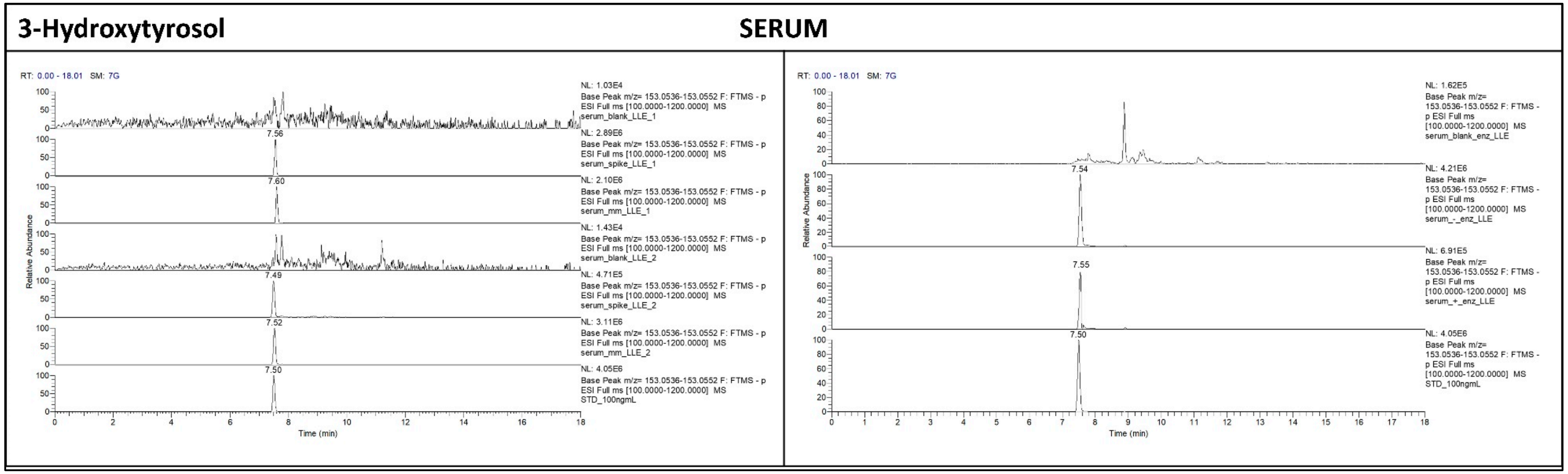

Figure S7b. (continued)

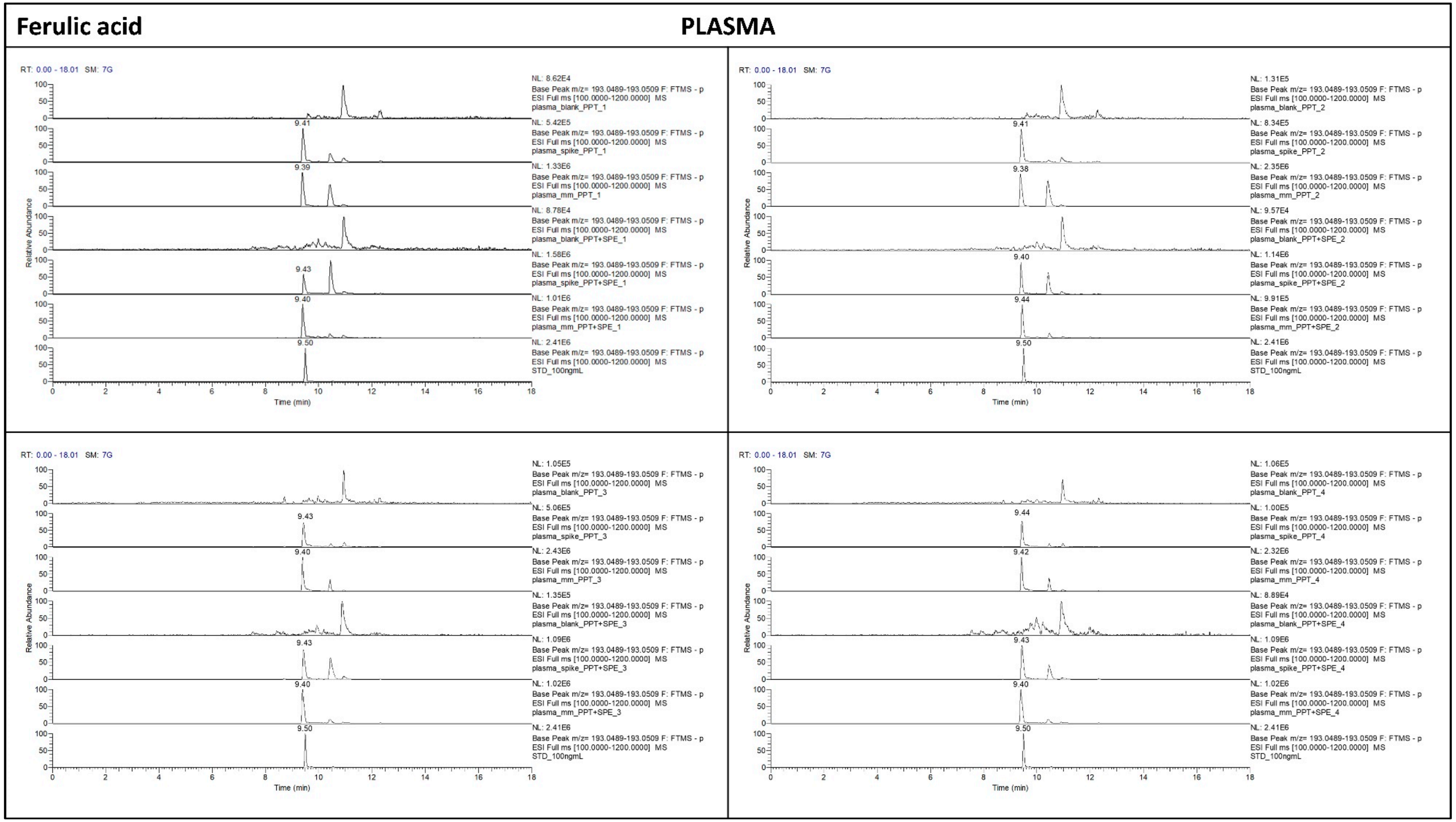

Figure S8a. Extracted ion chromatograms (XICs) of ferulic acid (9.50 min) for each pre-treatment method tested for plasma samples.

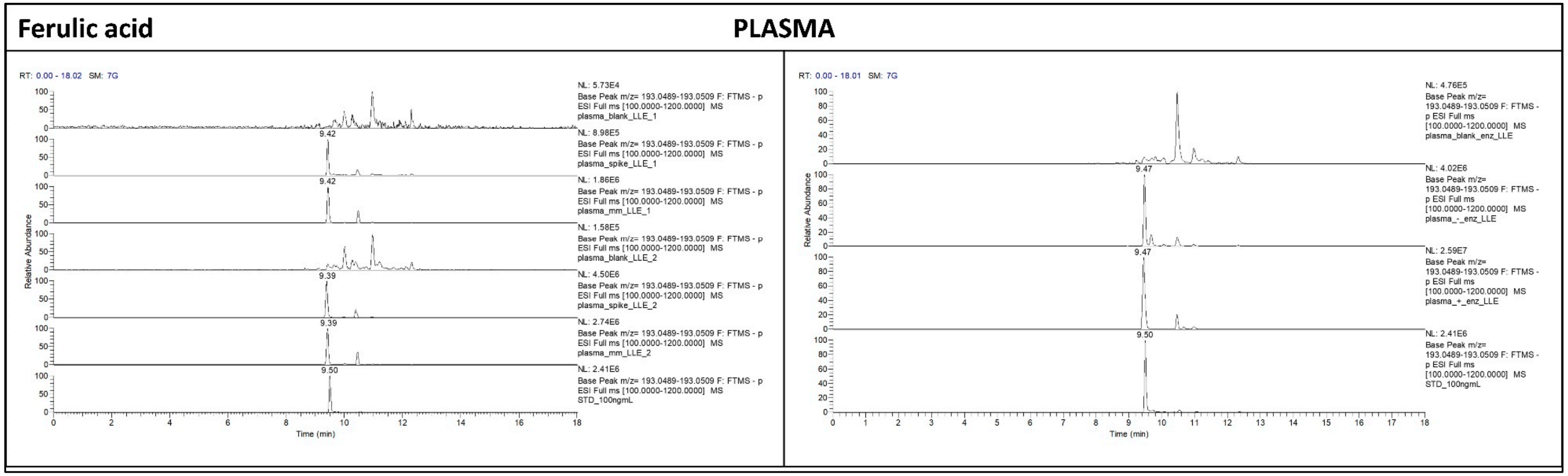

Figure S8a. (continued)

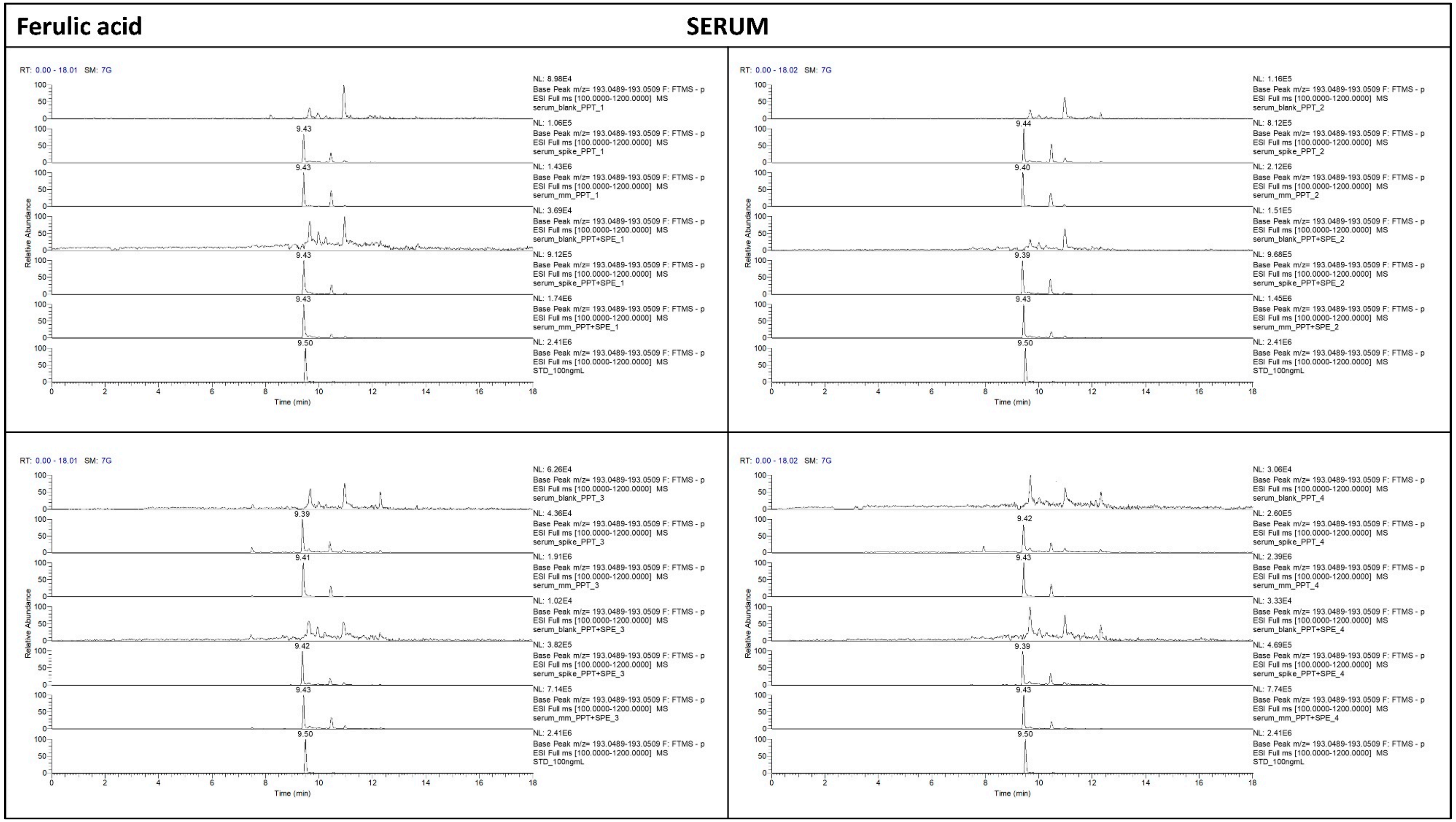

Figure S8b. Extracted ion chromatograms (XICs) of ferulic acid (9.50 min) for each pre-treatment method tested for serum samples.

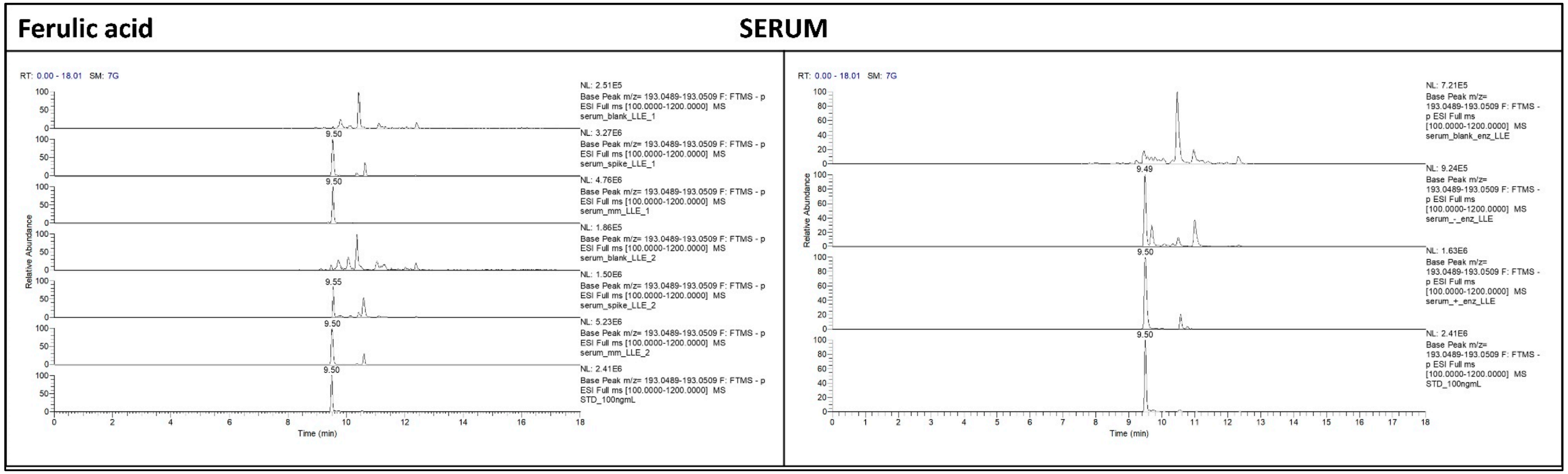

Figure S8b. (continued)

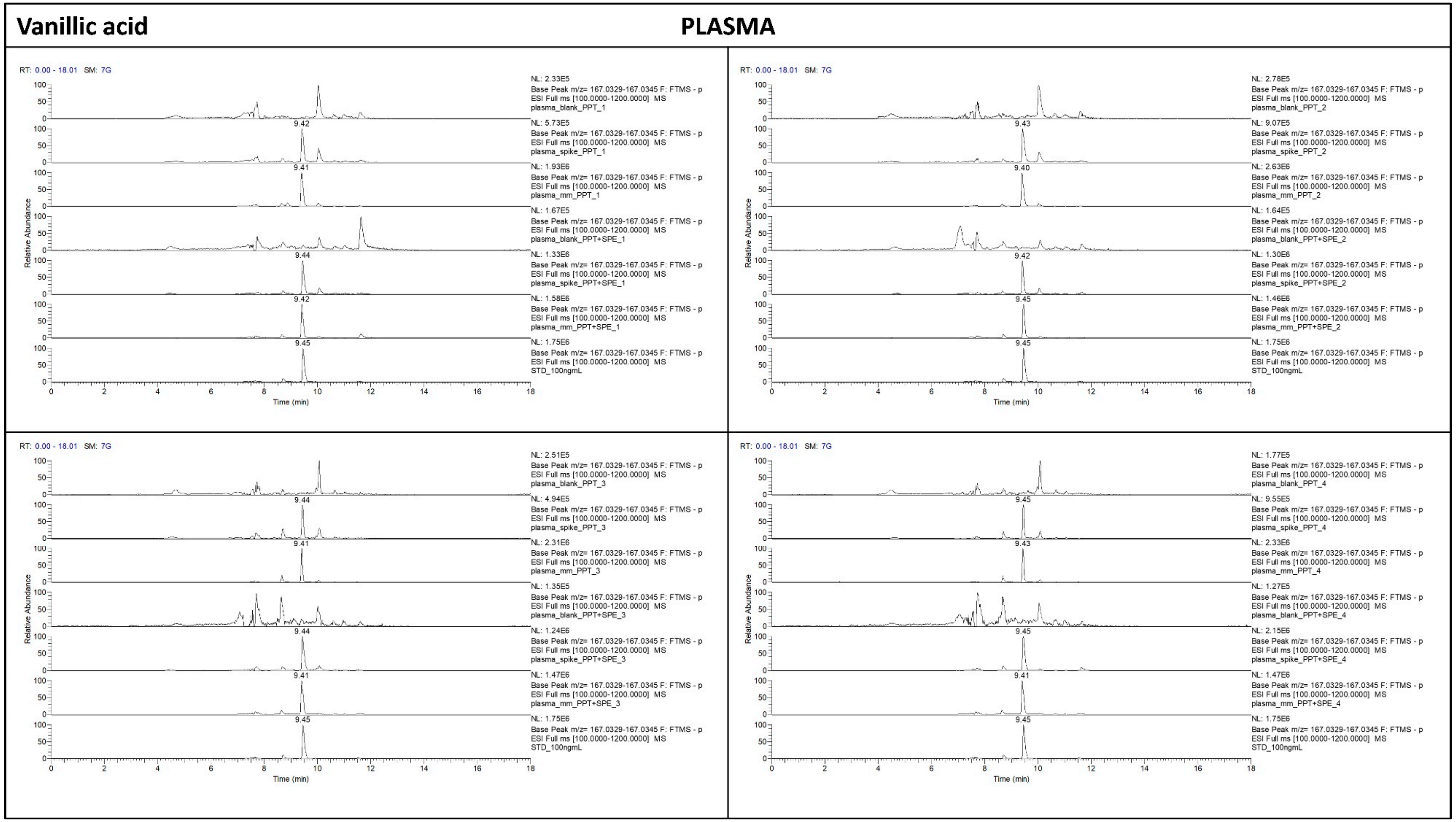

Figure S9a. Extracted ion chromatograms (XICs) of vanillic acid (9.50 min) for each pre-treatment method tested for plasma samples.

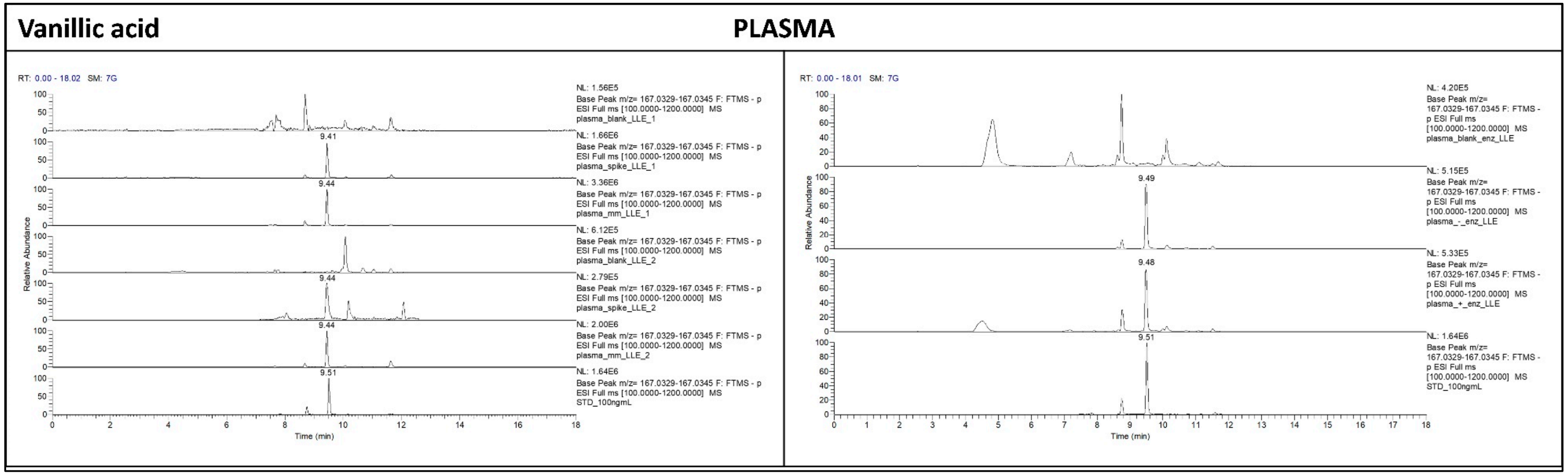

Figure S9a. (continued)

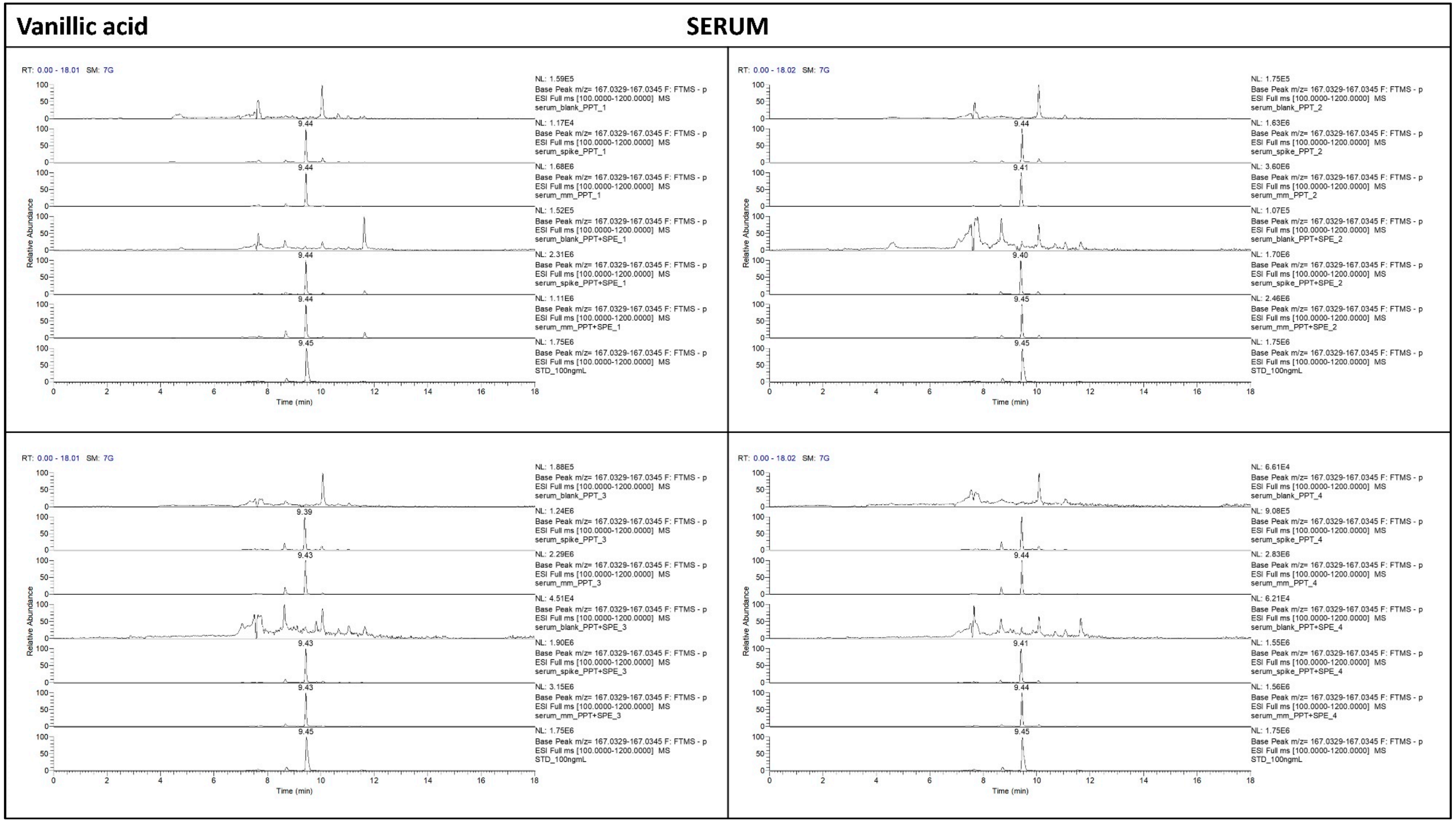

Figure S9b. Extracted ion chromatograms (XICs) of vanillic acid (9.50 min) for each pre-treatment method tested for serum samples.

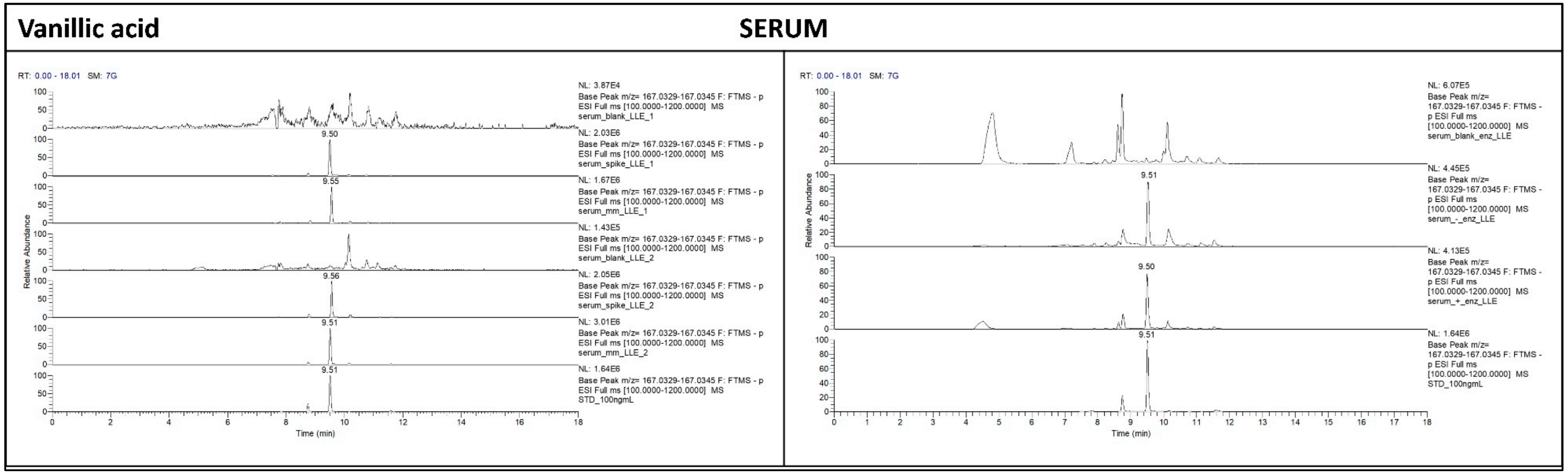

Figure S9b. (continued)

Table S2. Calibration curves obtained in serum matrix, linear ranges, limits of detection (LOD), limits of quantitation (LOQ) and matrix effects (%ME) of each analyte.

Analyte	Matrix-matched Calibration Curve (serum)	R^2	Range (ng/mL)	Matrix Effect (%ME)	LOD/LOQ (ng/mL, serum)
		**Isoflavones**			
Daidzein	y = 0.2595x + 2.6735	0.9931	0.5-300	-14	0.2/ 0.7
Formononetin	y = 0.0982x + 0.4012	0.9959	0.5-300	-19	0.1/ 0.3
Genistein	y = 0.1907x + 1.3104	0.9941	0.5-300	1	1.1/ 3.2
		**Flavanones**			
Hesperetin	y = 0.0825x + 0.4625	0.9952	0.5-300	-18	1.1/ 3.2
Naringenin	y = 0.1212x + 0.7799	0.9967	0.5-300	-18	0.9/ 2.7
		**Flavones**			
Apigenin	y = 0.1379x + 1.1436	0.9932	0.5-300	-32	0.9/ 2.9
Chrysin	y = 0.1606x + 0.3186	0.9909	0.5-300	33	0.6/ 1.9
Luteolin	y = 0.0937x + 0.4716	0.9932	0.5-300	-48	0.2/ 0.6
		**Flavonols**			
Isorhamnetin	y = 0.0216x + 0.0998	0.9948	0.5-300	-39	0.8/ 2.5
Kaempferol	y = 0.0926x + 0.3899	0.9966	0.5-300	-48	0.9/ 2.9
Quercetin	y = 0.0367x + 0.0093	0.9990	0.5-300	-37	0.5/ 1.6
		**Flavan-3-ols**			
Catechin	y = 0.0184x + 0.0651	0.9964	0.5-300	-35	0.7/ 2.1
Epicatechin	y = 0.0189x + 0.0597	0.9986	0.5-300	-32	0.8/ 2.5
Epicatechin gallate	y = 0.0032x + 0.0044	0.9981	0.5-300	-30	3.2/ 9.7
Epigallocatechin	y = 0.0147x - 0.0221	0.9980	0.5-300	-24	0.7/ 2.2
Epigallocatechin gallate	y = 0.0014x - 0.0084	0.9977	0.5-300	-90	0.4/ 1.3
		**Flavonol glycosides**			
Isoquercetin	y = 0.0028x + 0.0086	0.9979	0.5-300	-40	0.2/ 0.6
Rutin	y = 0.0007x + 0.0038	0.9981	5-300	-36	0.9/ 2.7
		**Stilbenes**			
trans-Resveratrol	y = 0.0226x + 0.1796	0.9907	0.5-300	9	0.5/ 1.6
E-Viniferin	y = 0.0018x + 0.0045	0.9979	0.5-300	-18	0.8/ 2.4
Piceid	y = 0.0033x + 0.0150	0.9968	0.5-300	-20	1.1/ 3.2
		**Phenylethanoids**			
3-Hydroxytyrosol	y = 0.0494x + 0.3187	0.9900	0.5-300	-6	0.8/ 2.4
Oleuropein	y = 0.0018x + 0.0103	0.9967	1-300	-53	1.1/ 3.3
		**Cinnamic acid and derivatives**			
trans-Cinnamic acid	y = 0.0008x + 0.0019	0.9953	5-300	-47	0.8/ 2.3
Caffeic acid	y = 0.0919x - 0.0970	0.9975	0.5-300	-57	1.6/ 4.9

Chlorogenic acid	y = 0.0070x + 0.0392	0.9943	0.5-300	-29	0.7/ 2
o-Coumaric acid	y = 0.0442x - 0.0671	0.9982	0.5-300	26	0.9/ 2.9
p-Coumaric acid	y = 0.1147x - 0.1141	0.9980	0.5-300	6	1.1/ 3.2
Ferulic acid	y = 0.0279x - 0.0277	0.9979	0.5-300	-9	0.9/ 2.6
Neochlorogenic acid	y = 0.0035x + 0.0036	0.9979	0.5-300	-10	0.8/ 2.4
Sinapic acid	y = 0.0258x + 0.0095	0.9960	0.5-300	7	0.9/ 2.8
		**Benzoic acid derivatives**			
Gallic acid	y = 0.0357x - 0.1406	0.9979	0.5-300	-67	0.5/ 1.6
Syringic acid	y = 0.0050x - 0.0115	0.9975	5-300	-43	1.1/ 3.2
Vanillic acid	y = 0.0028x + 0.0003	0.9980	1-300	-28	3.1/ 9.3

Table S3. Accuracy (error, RE%) and precision data (RSD%) of the analytes in spiked serum matrix (n = 6).

Analytes	Concentration (ng/mL)	Accuracy RE (%)	Intra-day RSD (%)	Inter-day RSD (%)	Recovery (% mean ± SD)
	**Isoflavones**				
Daidzein	1	4.2	7.0	10.7	75 ± 6
	20	1.3	5.8	3.7	71 ± 1
	60	8.8	6.3	7.2	78 ± 8
	160	1.3	6.2	4.4	85 ± 11
Formononetin	1	1.8	0.3	1.3	81 ± 9
	20	4.4	6.0	4.0	81 ± 4
	60	2.0	3.8	6.3	93 ± 8
	160	-8.4	4.7	7.2	79 ± 11
Genistein	1	-10.8	7.5	6.4	81 ± 7
	20	-7.5	8.0	6.8	72 ± 3
	60	-0.2	4.3	4.4	78 ± 6
	160	0.1	6.6	4.3	89 ± 6
	**Flavanones**				
Hesperetin	10	-0.7	3.3	3.2	74 ± 4
	40	2.0	5.1	4.7	70 ± 6
	80	5.7	1.6	5.5	64 ± 5
	160	-3.6	3.8	5.6	74 ± 4
Naringenin	10	8.1	1.6	7.2	66 ± 4
	40	1.1	3.7	4.7	66 ± 7
	80	4.9	5.1	6.0	70 ± 7
	160	-7.9	3.9	7.7	72 ± 6
	**Flavones**				
Apigenin	10	-4.0	7.1	4.9	66 ± 5
	40	-7.4	5.4	6.3	74 ± 6
	80	-8.3	6.4	4.5	83 ± 5

	160	-1.3	5.5	4.0	74 ± 3
Chrysin	10	6.2	3.4	3.9	81 ± 7
	40	-7.5	7.1	13.5	77 ± 13
	80	-9.3	5.2	7.2	78 ± 8
	160	-7.5	3.0	8.2	75 ± 13
Luteolin	1	-5.7	12.8	10.0	66 ± 8
	40	-0.2	1.6	2.2	72 ± 5
	80	5.7	4.7	9.9	61 ± 4
	160	-3.8	3.7	6.0	71 ± 2
**Flavonols**					
Isorhamnetin	10	1.4	10.9	7.4	64 ± 4
	40	5.2	7.3	6.6	69 ± 5
	80	0.3	5.7	6.6	70 ± 3
	160	-3.7	3.2	4.0	73 ± 5
Kaempferol	10	5.5	2.6	5.4	61 ± 9
	40	-1.3	3.0	4.3	67 ± 2
	80	-2.3	4.4	4.9	61 ± 6
	160	-5.6	4.5	5.1	75 ± 7
Quercetin	10	5.1	6.1	8.2	69 ± 6
	40	-2.1	7.0	5.2	72 ± 6
	80	0.3	1.8	5.2	68 ± 5
	160	-0.9	4.4	3.4	70 ± 9
**Flavan-3-ols**					
Epicatechin	10	5.2	5.4	3.7	69 ± 4
	40	2.4	14.3	12.1	73 ± 9
	80	-0.8	5.0	6.6	64 ± 4
	100	-7.9	4.7	7.2	72 ± 6
Epigallocatechin	10	-11.2	1.5	4.0	53 ± 3
	40	1.6	5.1	4.7	82 ± 6
	80	-1.0	2.0	4.9	78 ± 4
	160	4.0	5.3	4.2	83 ± 20
Epigallocatechin gallate	10	1.3	8.0	9.0	76 ± 6
	40	-7.7	0.7	4.1	75 ± 5
	80	0.7	7.8	7.2	92 ± 6
	160	-7.3	0.5	7.5	90 ± 5
Catechin	10	-1.2	8.4	5.7	71 ± 2
	40	3.0	1.7	10.0	77 ± 11
	80	9.3	5.4	9.5	64 ± 9
	160	-3.7	6.5	7.9	78 ± 9
Epicatechin gallate	10	3.2	2.4	4.6	83 ± 1
	40	-11.0	11.9	8.0	88 ± 10
	80	-0.9	5.8	7.5	76 ± 5
	160	-10.9	5.8	6.9	92 ± 11
**Flavonol glycosides**					
Isoquercetin	1	-3.5	7.5	6.0	53 ± 2

	20	0.2	3.4	3.4	60 ± 4
	60	-2.2	4.9	4.8	71 ± 7
	160	2.3	2.6	2.8	80 ± 6
Rutin	10	3.8	3.1	4.9	56 ± 6
	40	-0.3	3.0	5.1	86 ± 10
	80	-0.8	1.1	6.3	72 ± 9
	160	-1.4	3.3	3.3	85 ± 8
**Stilbenes**					
trans-Resveratrol	10	4.0	4.6	9.4	59 ± 6
	40	2.0	9.6	9.7	56 ± 6
	80	1.2	0.9	7.0	56 ± 5
	160	-0.3	0.4	1.7	48 ± 6
E-Viniferin	10	-1.2	1.5	3.6	73 ± 3
	40	1.4	3.0	6.5	70 ± 6
	80	-1.4	5.3	4.1	81 ± 4
	160	-0.6	1.9	3.4	82 ± 7
Piceid	10	0.0	8.5	6.0	62 ± 6
	40	7.2	2.1	5.7	83 ± 13
	80	-1.0	4.3	4.4	73 ± 5
	160	0.3	5.0	3.7	73 ± 22
**Phenylethanoids**					
3-Hydroxytyrosol	10	4.4	8.3	11.2	68 ± 9
	40	6.6	3.0	6.8	77 ± 7
	80	-3.8	4.1	6.4	68 ± 7
	160	1.8	1.2	5.0	70 ± 7
Oleuropein	10	2.9	6.8	5.8	84 ± 4
	40	-0.3	8.3	5.9	81 ± 2
	80	0.6	2.0	2.3	79 ± 6
	160	1.1	1.1	2.3	81 ± 8
**Cinnamic acid and derivatives**					
Caffeic acid	10	3.6	1.8	10.2	86 ± 8
	40	-7.7	8.5	8.4	80 ± 10
	80	-2.7	6.1	7.5	67 ± 5
	160	-1.4	4.8	3.4	81 ± 5
trans-Cinnamic acid	10	3.4	5.6	6.5	42 ± 5
	40	2.0	1.6	7.2	40 ± 5
	80	2.3	1.5	4.0	38 ± 4
	160	-0.6	2.8	1.9	46 ± 12
Chlorogenic acid	10	-3.0	4.5	7.4	55 ± 6
	40	-0.8	1.5	3.8	50 ± 5
	80	-0.6	1.6	4.4	65 ± 9
	160	0.2	2.6	2.1	56 ± 4
o-Coumaric acid	10	-1.6	3.9	4.8	87 ± 8
	40	-3.8	6.0	6.7	82 ± 1
	80	-1.7	3.4	6.0	76 ± 9

	160	-3.4	7.8	6.3	77 ± 7
p-Coumaric acid	10	5.2	5.1	8.5	72 ± 11
	40	-4.4	5.5	6.9	83 ± 7
	80	-7.3	3.5	6.0	81 ± 6
	160	-1.4	8.5	5.7	87 ± 10
Ferulic acid	10	14.9	0.8	10.6	73 ± 12
	40	-0.1	8.8	7.4	67 ± 9
	80	-2.0	6.0	7.0	71 ± 9
	160	-7.0	3.3	7.4	84 ± 14
Neochlorogenic acid	10	3.7	0.6	4.6	28 ± 2
	40	-4.1	8.4	6.9	45 ± 5
	80	-8.0	4.9	5.8	29 ± 7
	160	1.6	2.6	1.9	41 ± 8
Sinapic acid	10	4.9	4.2	5.6	78 ± 6
	40	-2.3	7.4	9.1	83 ± 5
	80	0.2	1.0	1.6	71 ± 7
	160	-1.2	2.3	3.2	78 ± 8
**Benzoic acid derivatives**					
Gallic acid	1	3.0	4.2	5.3	36 ± 6
	40	-7.1	7.7	5.4	32 ± 3
	80	-6.7	2.1	2.1	33 ± 1
	160	5.3	1.4	2.1	42 ± 9
Syringic acid	10	3.0	3.4	6.3	46 ± 49
	40	-0.1	4.6	4.1	12 ± 3
	80	0.9	0.2	1.3	27 ± 4
	160	0.5	1.4	1.9	27 ± 3
Vanillic acid	10	4.4	3.4	6.8	66 ± 9
	40	-3.8	6.9	5.6	59 ± 5
	80	-1.1	3.2	5.3	84 ± 8
	160	-0.2	3.1	3.3	80 ± 8

Table S4. Stability of analytes in serum samples under various storage conditions (n = 3).

Analyte	Concentration (ng/mL)	Auto sampler temperature (4°C) after 24 h	After one freeze-thaw cycle	Long-term stability (2 months, stored at -40°C)
**Isoflavones**				
Daidzein	10	9.6 ± 0.3 (3.6)	10.3 ± 1 (9.4)	10.1 ± 1 (10)
	80	88.1 ± 6.6 (7.4)	82.7 ± 4.3 (5.1)	84.1 ± 4.7 (5.6)
Formononetin	10	10 ± 0.4 (4.1)	10.1 ± 0.2 (2.1)	10.2 ± 0.1 (1.4)
	80	82.3 ± 10.7 (13)	83.3 ± 5.6 (6.7)	79.2 ± 5.4 (6.8)
Genistein	10	10.4 ± 0.8 (7.6)	10 ± 1 (9.5)	10.1 ± 1.1 (11.1)
	80	81.7 ± 2.3 (2.8)	78.5 ± 3 (3.9)	78.1 ± 4.1 (5.2)
**Flavanones**				
Hesperetin	10	10.2 ± 0.5 (4.5)	10.2 ± 0.3 (3.2)	10.3 ± 0.1 (0.7)
	80	86.8 ± 5.7 (6.6)	83.1 ± 1.4 (1.7)	85.2 ± 7 (8.2)
Naringenin	10	10.1 ± 0.8 (7.8)	9.8 ± 0.8 (8.3)	9.9 ± 0.7 (7)
	80	81.5 ± 7.3 (9)	79.3 ± 5.2 (6.5)	81.3 ± 2.2 (2.7)
**Flavones**				
Apigenin	10	9.4 ± 0.1 (0.9)	9.6 ± 0.7 (6.9)	9.9 ± 0.5 (4.6)
	80	73.1 ± 4.3 (5.9)	75.6 ± 1.6 (2.1)	74.4 ± 2 (2.7)
Chrysin	10	10.3 ± 0.4 (3.4)	10.4 ± 0.6 (5.3)	10.3 ± 0.3 (3)
	80	79.7 ± 4.1 (5.1)	74.3 ± 3.5 (4.7)	75.2 ± 8 (10.7)
Luteolin	10	10.4 ± 0.5 (4.5)	10.4 ± 0.1 (0.7)	10.6 ± 0.3 (2.7)
	80	82.2 ± 3.6 (4.4)	80.4 ± 6.7 (8.4)	73.5 ± 6.8 (9.3)
**Flavonols**				
Isorhamnetin	10	10.4 ± 0.5 (4.3)	9.9 ± 0.8 (8.1)	10.4 ± 1.2 (11.1)
	80	81.6 ± 7 (8.6)	80.7 ± 5.2 (6.4)	75.2 ± 1.4 (1.9)
Kaempferol	10	10.2 ± 0.2 (2.4)	10 ± 0.8 (8)	10 ± 0.7 (6.7)
	80	79.6 ± 4.9 (6.2)	80.9 ± 2.7 (3.3)	76.9 ± 4.2 (5.5)
Quercetin	10	9.7 ± 1 (10.7)	9.5 ± 0.8 (7.9)	10.3 ± 0.7 (6.4)
	80	80.1 ± 2.6 (3.2)	76.4 ± 5.1 (6.7)	77.2 ± 5.7 (7.3)
**Flavan-3-ols**				
Catechin	10	10.1 ± 0.6 (6.1)	9.4 ± 0.2 (2.4)	9.9 ± 0.7 (7.3)
	80	81.5 ± 10.3 (12.6)	77.2 ± 7.3 (9.4)	80.9 ± 6.6 (8.2)
Epicatechin	10	10.5 ± 0.2 (2.2)	10.6 ± 0.5 (4.9)	10.8 ± 0.6 (5.1)
	80	79.1 ± 7.7 (9.8)	74.8 ± 4 (5.3)	78.8 ± 4.5 (5.8)
Epicatechin gallate	10	10.5 ± 0.6 (5.3)	10.9 ± 0.4 (3.5)	10.4 ± 0.6 (6)
	80	75.1 ± 7.5 (10)	77.4 ± 4.1 (5.3)	74.3 ± 7.5 (10.1)
Epigallocatechin	10	8.8 ± 0.5 (5.5)	8.8 ± 0.5 (5.4)	9.2 ± 0.1 (1.5)
	80	80.2 ± 3.4 (4.3)	79.3 ± 1.5 (1.9)	78.4 ± 5.8 (7.4)
Epigallocatechin gallate	10	11 ± 0.9 (8.6)	10.8 ± 1.2 (11.1)	10.6 ± 1 (9.3)
	80	78.2 ± 2.1 (2.7)	81.8 ± 5.5 (6.7)	74.8 ± 3.6 (4.8)
**Flavonol glycosides**				
**Isoquercetin**	10	10.9 ± 0.1 (1.2)	10.5 ± 0.4 (3.4)	10.8 ± 0.6 (5.2)
	80	82.3 ± 7.5 (9.1)	81.3 ± 6 (7.4)	76.5 ± 2.7 (3.6)

**Rutin**	10	10.1 ± 0.1 (1.4)	9.8 ± 0.5 (5.6)	10.1 ± 0.8 (7.5)
	80	84.6 ± 6.9 (8.1)	85.1 ± 6.8 (7.9)	81 ± 2.5 (3.1)
**Stilbenes**				
trans-Resveratrol	10	9.2 ± 0.6 (7.1)	9.4 ± 1.2 (12.8)	9.5 ± 0.9 (9)
	80	80.5 ± 4 (5)	80.1 ± 3.9 (4.9)	72.8 ± 7.1 (9.8)
E-Viniferin	10	10.3 ± 0.5 (4.5)	10.3 ± 0.5 (4.8)	10.2 ± 0.1 (1.3)
	80	82.7 ± 1.8 (2.2)	78.5 ± 2.2 (2.8)	79.7 ± 3.5 (4.4)
Piceid	10	9.8 ± 0.7 (7.2)	10.3 ± 0.3 (2.5)	10.3 ± 0.5 (5.2)
	80	82.2 ± 3.7 (4.5)	81.5 ± 5.5 (6.7)	81.9 ± 1.2 (1.4)
**Phenylethanoids**				
3-Hydroxytyrosol	10	10.1 ± 1.4 (13.4)	9 ± 0.6 (6.7)	9.7 ± 1.5 (15.6)
	80	83.3 ± 5.1 (6.2)	80.2 ± 2.6 (3.2)	79.9 ± 5.7 (7.1)
Oleuropein	10	10.6 ± 0.8 (7.3)	10.5 ± 0.4 (3.8)	10.9 ± 0.5 (4.3)
	80	81 ± 2.3 (2.9)	81.7 ± 2.2 (2.7)	80.4 ± 1.6 (2)
**Cinnamic acid and derivatives**				
trans-Cinnamic acid	10	9.6 ± 0.4 (4.4)	9.5 ± 0.4 (3.8)	10 ± 0.9 (9)
	80	83.3 ± 0 (0)	80.7 ± 0.3 (0.4)	79.7 ± 4.5 (5.7)
Caffeic acid	10	9.1 ± 1.2 (12.9)	9.7 ± 1 (10.7)	9.9 ± 0.7 (7.1)
	80	75 ± 8.9 (11.9)	74.8 ± 0.6 (0.8)	72.1 ± 5.6 (7.8)
Chlorogenic acid	10	10.2 ± 0.8 (7.5)	10.6 ± 0.6 (6.1)	10.6 ± 0.9 (8.9)
	80	77.4 ± 3.6 (4.7)	75.7 ± 4 (5.3)	78.1 ± 4.1 (5.2)
o-Coumaric acid	10	9.5 ± 0.5 (4.9)	10 ± 0.2 (2.5)	9.8 ± 0.7 (7.2)
	80	78.7 ± 5.4 (6.9)	76.8 ± 5.5 (7.2)	73.3 ± 3 (4.1)
p-Coumaric acid	10	9.6 ± 0.6 (6.5)	9.5 ± 0.6 (6.2)	9.7 ± 1.2 (12.6)
	80	76.3 ± 5.5 (7.2)	75 ± 5.9 (7.9)	70.6 ± 1 (1.4)
Ferulic acid	10	10.1 ± 1.1 (10.8)	10.1 ± 1.2 (12.2)	10.2 ± 1.2 (11.8)
	80	77.8 ± 7.6 (9.8)	77.7 ± 4 (5.2)	72 ± 2.6 (3.6)
Neochlorogenic acid	10	10.4 ± 0.1 (1.4)	10.7 ± 0.4 (3.7)	10.8 ± 0.7 (6.1)
	80	78 ± 2.2 (2.8)	73.1 ± 3.4 (4.7)	73.3 ± 6.2 (8.5)
Sinapic acid	10	9.8 ± 0.3 (3.1)	9.9 ± 0.7 (7.4)	10.3 ± 0.7 (6.7)
	80	79.4 ± 1 (1.3)	79.2 ± 1.6 (2.1)	78.4 ± 0.9 (1.1)
**Benzoic acid derivatives**				
Gallic acid	10	9.3 ± 1.1 (11.6)	9.6 ± 1.1 (11)	10.4 ± 0.4 (4.2)
	80	74.6 ± 1.9 (2.6)	74.6 ± 1.7 (2.3)	74.9 ± 1.7 (2.3)
Syringic acid	10	9.4 ± 0.5 (4.8)	9.8 ± 0.7 (6.9)	10.2 ± 0.5 (4.8)
	80	79.9 ± 0.8 (1)	79.4 ± 1.3 (1.7)	79.8 ± 1.3 (1.6)
Vanillic acid	10	10 ± 0.7 (7)	9.5 ± 0.5 (5.8)	10.1 ± 0.8 (8.3)
	80	76.3 ± 4.8 (6.3)	78.6 ± 4.2 (5.3)	75.2 ± 4.5 (5.9)

Table S5

Polar phenol content of Corinthian currant and rat food (mg/100g).

Polar Phenol	Corinthian Currant	Rat Food
**Isoflavones**		
Daidzein	n.d.	111.6 ± 21.6
Formononetin	n.d.	6.1 ± 1.4
Genistein	0.2 ± 0.1	286.3 ± 46.7
**Flavanones**		
Hesperetin	0.6 ± 0	1.2 ± 0.3
Naringenin	2.2 ± 0.3	2.4 ± 0.8
**Flavones**		
Apigenin	n.d.	30.9 ± 3.3
Luteolin	1.14 ± 0.16	27.4 ± 8
Chrysin	0.15 ± 0.04	n.d.
**Flavonols**		
Isorhamnetin	27.3 ± 0.8	0.9 ± 0.2
Kaempferol	35 ± 4.8	12.1 ± 2
Quercetin	172.79 ± 19.38	4.1 ± 1.2
**Flavonol glycosides**		
Isoquercetin	479.61 ± 70.81	3.1 ± 1.3
Rutin	118.83 ± 13.91	11 ± 2.7
**Stilbenes**		
trans-Resveratrol	3.3 ± 0.4	n.d.
E-Viniferin	3.5 ± 0.2	n.d.
Piceid	27.9 ± 3.8	8.9 ± 1.3
**Cinnamic acid derivatives**		
Caffeic acid	52.1 ± 1.5	19.6 ± 2.4
Ferulic acid	9.9 ± 1.3	58.6 ± 14.8
p-Coumaric acid	10.9 ± 1.1	76.4 ± 12.5
**Benzoic acid derivatives**		
Gallic acid	193.1 ± 22.1	4.4 ± 1.5
Syringic acid	122.2 ± 18.7	33.4 ± 5
Vanillic acid	286.2 ± 45.1	39.5 ± 6.4
